# A Parallel Distributed-Memory Particle Method Enables Acquisition-Rate Segmentation of Large Fluorescence Microscopy Images

**DOI:** 10.1371/journal.pone.0152528

**Published:** 2016-04-05

**Authors:** Yaser Afshar, Ivo F. Sbalzarini

**Affiliations:** 1 Chair of Scientific Computing for Systems Biology, Faculty of Computer Science, Technische Universität Dresden, 01187 Dresden, Germany; 2 Max Planck Institute of Molecular Cell Biology and Genetics, 01307 Dresden, Germany; 3 MOSAIC Group, Center for Systems Biology Dresden, 01397 Dresden, Germany; University of Cambridge, UNITED KINGDOM

## Abstract

Modern fluorescence microscopy modalities, such as light-sheet microscopy, are capable of acquiring large three-dimensional images at high data rate. This creates a bottleneck in computational processing and analysis of the acquired images, as the rate of acquisition outpaces the speed of processing. Moreover, images can be so large that they do not fit the main memory of a single computer. We address both issues by developing a distributed parallel algorithm for segmentation of large fluorescence microscopy images. The method is based on the versatile Discrete Region Competition algorithm, which has previously proven useful in microscopy image segmentation. The present distributed implementation decomposes the input image into smaller sub-images that are distributed across multiple computers. Using network communication, the computers orchestrate the collectively solving of the global segmentation problem. This not only enables segmentation of large images (we test images of up to 10^10^ pixels), but also accelerates segmentation to match the time scale of image acquisition. Such acquisition-rate image segmentation is a prerequisite for the smart microscopes of the future and enables online data compression and interactive experiments.

## Introduction

Modern fluorescence microscopes with high-resolution cameras are capable of acquiring large images at a fast rate. Data rates of 1 GB/s are common with CMOS cameras, and the three-dimensional (3D) image volumes acquired by light-sheet microscopy [1] routinely exceed tens of gigabytes per image, and tens of terabytes per time-lapse experiment [2–4]. This defines new challenges in handling, storing, and analyzing the image data, as image acquisition outpaces analysis capabilities.

Ideally, the images are analyzed during acquisition with analysis times that are smaller than the time until the next image is acquired. This “real-time” image analysis not only alleviates the data bottleneck, but is also a prerequisite for smart microscopes that optimize the acquisition of the next image based on the contents of the current image [5]. Real-time segmentation also enables interactive experiments where, e.g., optical manipulation and tracking become feasible in a developing embryo [6].

Real-time, or more precisely acquisition-rate, segmentation of large images is usually hindered by the memory requirements of the image data and the analysis algorithm. Segmenting an image requires about 5 to 10 times more memory than the raw image data [7–9]. This means that in order to segment a 30 GB 3D light-sheet microscopy image, one would need a computer with 150 to 300 GB of main memory. Image segmentation at acquisition rate has hence mainly been achieved for smaller images [10]. For example, segmenting a 2048 × 2048 × 400 pixel image of stained nuclei, which translates to about 3 GB file size at 16 bit depth, required more than 32 GB of main memory [10].

Acquisition-rate processing of large images has so far been limited to low-level image processing, such as filtering or blob detection. Pixel-by-pixel low-level processing has been accelerated by Olmedo, *et al.*, [11] using CUDA as a parallel programming tool on a graphics processing units (GPUs). In their work, pixel-wise operations are applied to many pixels simultaneously, rather than sequentially looping through pixels. While such GPU acceleration achieves high processing speeds and data rates, it is limited by the size of the GPU memory, which is in general smaller than the main memory. Another approach is to distribute different images to different computers. In a time-lapse sequence, every image can be sent to a different computer for processing. Using 100 computers, every computer has 100 frames time to finish processing its image, until it receives the next one. While this does not strictly fulfill the definition of acquisition-rate processing (e.g., it would not be useful for a smart microscope), it improves data throughput by pipelining. Galizia, *et al.*, [12] have demonstrated this in the parallel image processing library *GEnoa*, which runs on computer clusters using the Message Passing Interface (MPI) to distribute work, but it also runs on GPUs and GPU clusters. This library focuses on low-level image processing. Both GPU acceleration and embarrassingly parallel work-farming approaches are unable to provide acquisition-rate high-level image analysis of single large images or time series comprised of large images.

High-level image analysis in fluorescence microscopy is mostly concerned with image segmentation [13, 14]. In image segmentation, the task is to detect and delineate objects represented in the image. This is a high-level task, which cannot be done in a pixel-independent way. It also cannot be formulated as a shader or filter, rendering it hard to exploit the speed of GPUs. Finally, as outlined above, high-level image analysis of large images quickly exceeds the main memory of a single computer. This memory limitation can be overcome by sub-sampling the image, for example coarse-graining groups of pixels to *super-pixels*. This has been successfully used for acquisition-rate detection of nuclei and lineage tracking from large 3D images [6]. The generation of super-pixels only requires low-level operations, where the high-level analysis is done on the reduced data. While this effectively enables acquisition-rate high-level analysis, it does not provide single-pixel resolution and is somewhat limited to the specific application of lineage tracing.

Pixel-accurate high-level analysis of large images can be achieved by splitting each image into smaller sub-images and distributing them across multiple computers or memories, thus distributing the data and the work. The computers then work in parallel, each on its sub-image. They communicate over a network interconnect in order to collectively solve the same high-level image-analysis problem that a single computer would have solved. However, since the data are distributed, the solution is available faster, and arbitrarily large images can be accommodated by distributing across more computers. This is the hallmark of *distributed-memory parallelism*.

Here, we present a distributed-memory parallel implementation of a generic image segmentation algorithm. The present implementation scales to large images. Here we test images of size up to 8192 × 8192 × 256 = 1.7 ⋅ 10^10^ pixels, corresponding to 32 GB of data per image at 16 bit depth. Distributing an image across 128 computers enabled acquisition-rate segmentation of large light-sheet microscopy images of *Drosophila* embryos. The image-segmentation method implemented is *Discrete Region Competition* (DRC) [15], which is a general-purpose model-based segmentation method. It is not limited to nucleus detection or any other task, but solves generic image segmentation problems with pixel accuracy. The method is based on using computational particles to represent image regions. This particle-method character renders the computational cost of the method independent of the image size, since it only depends on the total contour length of the segmentation. Storing the information on particles effectively reduces the problem from 3D to 2D (or from 2D to 1D). Moreover, the particle nature of the method lends itself to distributed parallelism, as particles can be processed concurrently, even if pixels cannot. In terms of computational speed, DRC has been shown competitive with fast discrete methods from computer vision, such as multi-label graph-cuts [15, 16]. DRC has previously been demonstrated on 2D and 3D images using a variety of different image models, including piecewise constant, piecewise smooth, and deconvolving models [15].

The piecewise constant and piecewise smooth models are also available in the present distributed-memory parallel implementation. This makes available a state-of-the-art generic image segmentation toolbox for acquisition-rate analysis and analysis of large images that do not need to fit the memory of a single computer. The main challenge in parallelizing the DRC algorithm is to ensure global topological constraints on the image regions. These are required in order for regions to remain closed or connected. The main algorithmic contribution of the present work is hence to propose a novel distributed algorithm for the independent-sub-graph problem. The algorithmic solutions presented hereafter ensure that the final result computed is the same that would have been computed on a single computer, and that the network-communication overhead is kept to a minimum, hence ensuring scalability to large images.

Since each computer only stores its local sub-image, information needs to be communicated between neighboring sub-images in order to ensure global consistency of the solution. Since DRC is a particle method, we use the Parallel Particle Mesh (PPM) library [17–19] for work distribution and orchestration of the parallel communication. In the following, we briefly review DRC and then describe how it can be parallelized in a distributed-memory environment. We then present the main algorithmic contribution that made this possible: the distributed independent-sub-graph algorithm. We demonstrate correctness of the parallel implementation by comparing with the sequential reference implementation of DRC [15], as available in ITK [20]. We then benchmark the scalability and parallel efficiency of the new parallel implementation on synthetic images, where the correct solution is known. Finally, we showcase the use of the present implementation for acquisition-rate segmentation of light-sheet fluorescence microscopy images.

## Methods

Since the introduction of active contours [21], deformable models have extensively been used for image segmentation. They are characterized by a geometry representation and an evolution law [22]. Thorough reviews of deformable models can be found in Refs. [22, 23]. The geometry representation of the evolving contours in the image can be continuous or discrete, and in either case implicit (also called “geometric models”) or explicit (also called “parametric models”) [24].

### Review of Discrete Region Competition

**Fig 1:**
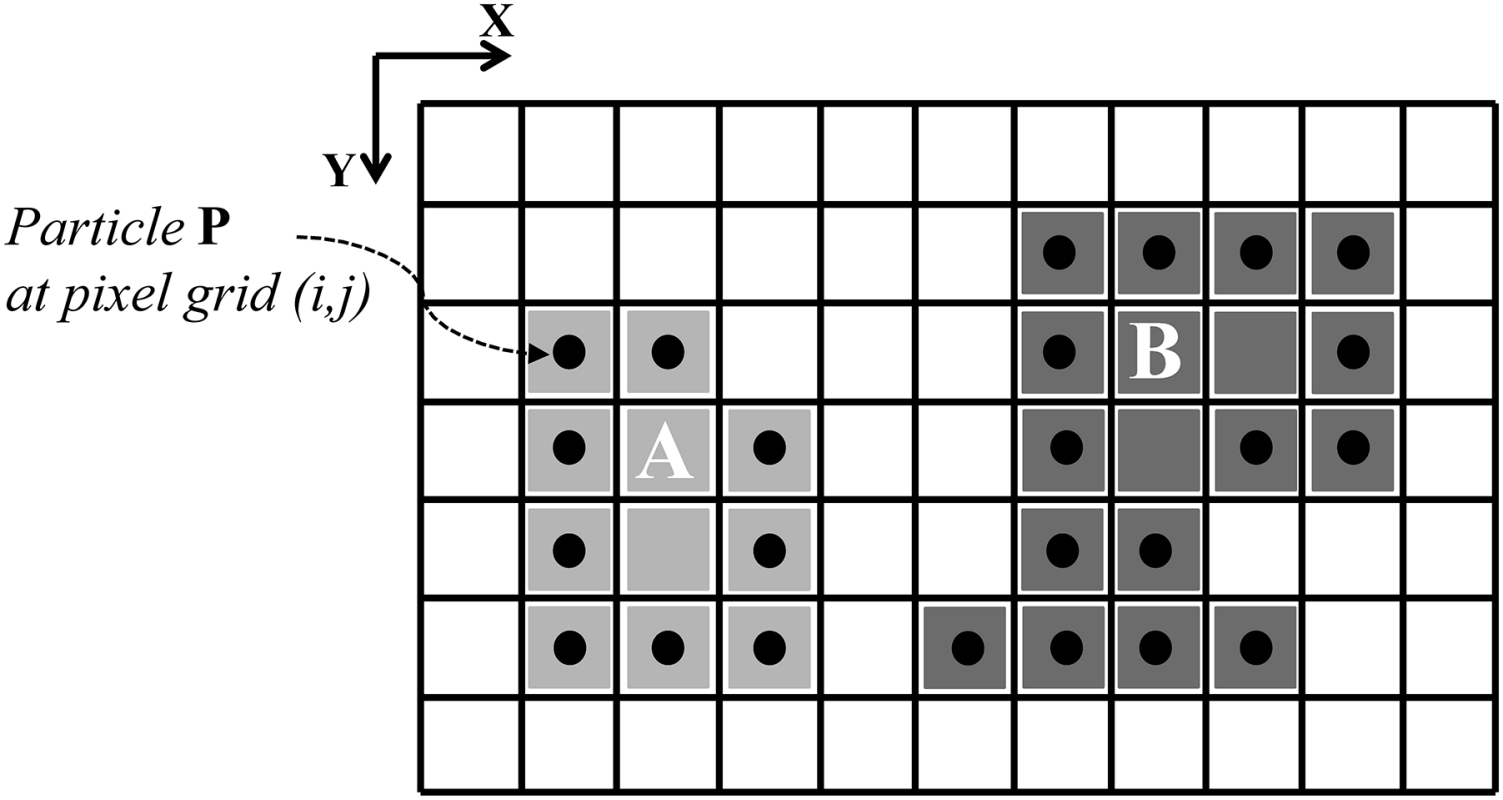
Illustration of 2 regions (A, light gray and B, dark gray) in a 2D digital image (grid). Pixels in the background region are white. Particles are shown as black filled circles. They represent the regions by marking their outlines. Shaded pixels without a particle are interior points of the respective region.

Inspired by discrete level-set methods [25], and motivated by the wish to delineate different objects in an image as individual regions, Cardinale *et al.* [15] presented a discrete deformable model where the contour is represented by computational particles placed on the pixel grid. This is illustrated in Fig 1 and provides a geometry representation that is both explicit and implicit [25]. During the iterative segmentation process, the particles migrate to neighboring pixels and hence deform the contour. This migration is driven by an energy-minimization flow. Additional topological constraints ensure that contours remain closed and/or connected. The algorithm is a discrete version of Region Competition [26], which converges to a locally optimal solution. It is called *Discrete Region Competition* (DRC), since particles from adjacent regions compete for ownership over pixels along common boundaries.

The algorithm partitions a digital image domain $\Omega \subset Z^{d}$ (the dimension *d* = 2 or 3) into a background (BG) region *X*_0_ and (*M* − 1) > 0 disjoint foreground (FG) regions *X*_*i*_, *i* = 1, ⋯, *M* − 1, bounded by contours Γ_*i*_, *i* = 1, ⋯, *M* − 1 [15].

FG regions are constrained to be connected sets of pixels. The void space around the FG regions is represented by a single BG region, which need not be connected. Connectivity in the FG regions is defined by a face-connected neighborhood, i.e., 4-connected in 2D and 6-connected in 3D. The BG region then has to be 8-connected in 2D and 18 or 26-connected in 3D [27]. Imposing the topological constraint that FG regions have to be connected sets of pixels regularizes the problem to the extent where the number of regions can be jointly estimated with their photometric parameters and contours [15].

The evolving contour is represented by computational particles as shown in Fig 1. The algorithm advances multiple particles simultaneously in a processing order that does not depend on particle indexing. This ensures convergence to a result that is independent of the order in which particles are visited. Connectedness of the evolving contours is ensured by topological control. The motion of the particles is driven by a discrete energy-minimization flow that locally minimizes the segmentation energy functional [15]:

$$\begin{array}{c} E=E_{\text{data}}+\lambda E_{\text{length}}+\alpha E_{\text{merge}}. \end{array}$$ (1)

Here, *λ* and *α* are regularization parameters trading off the weights of the contour-length and region-merging priors. The first term measures how well the current segmentation fits (or explains) the image. The specific forms of the three terms depend on the image model, imaging model, and object model used [15].

The above energy is minimized by approximate gradient descent. The gradient is approximated by the energy difference incurred by a particle move. Particles are then moved in order of descending energy reduction using a rank-based optimizer, hence ensuring that the result is independent of particle ordering [15]. Since regions may dynamically fuse and split during energy minimization, the algorithm is able to detect and segment a previously unknown and arbitrary number of regions.

**Fig 2:**
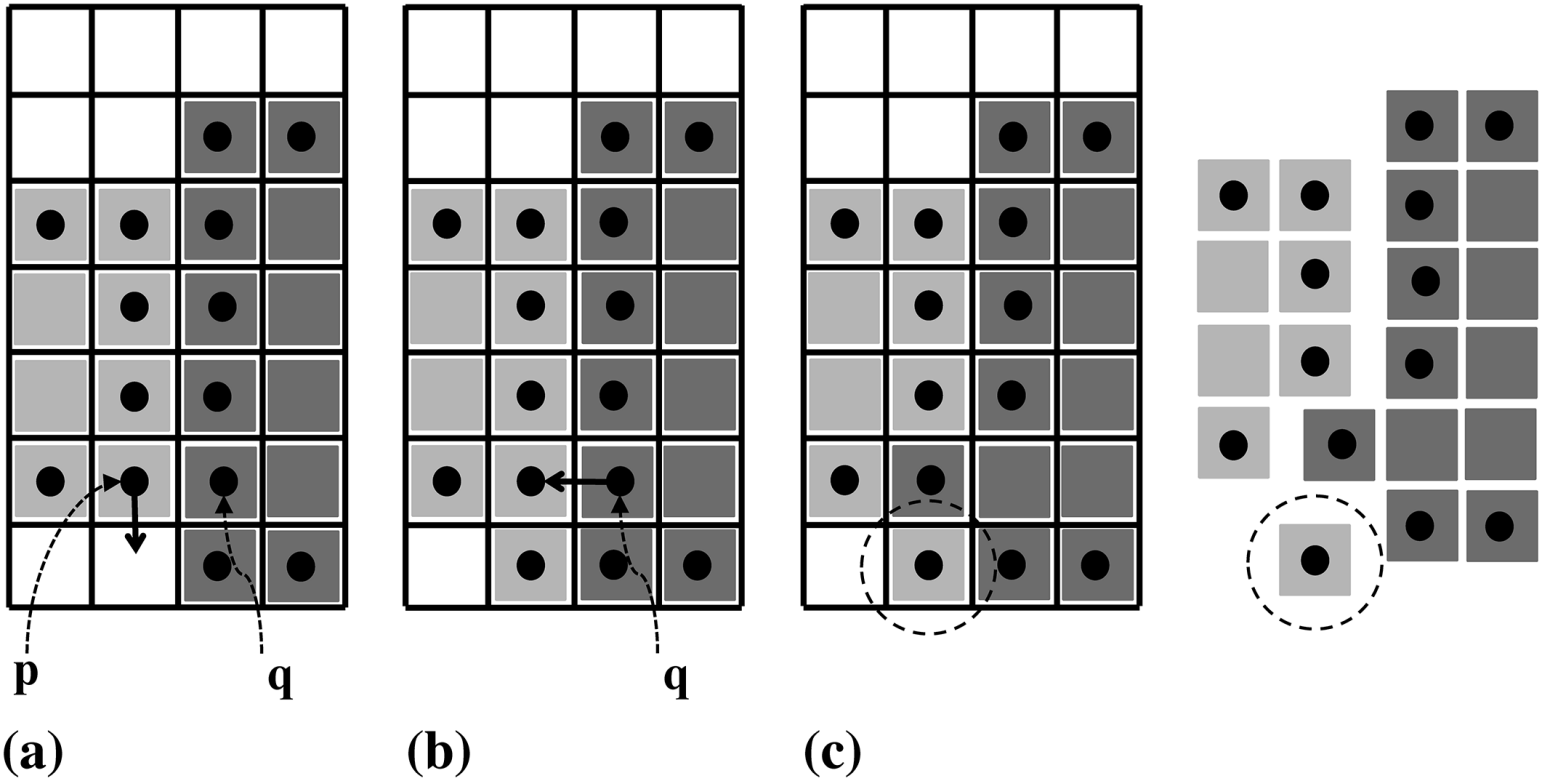
Illustration of causally dependent moves. Assume that the energetically most favorable moves are for particle **p** to move down (a) and particle **q** left (b). If both moves are executed, the light gray region is not connected any more, hence violating the topological constraint (c). The moves of the two particles hence causally depend on each other.

The algorithm starts from an initialization (frequently: local intensity maxima or an initial thresholding) and then refines the segmentation in iterations until no further improvement can be achieve by any particle move. In each iteration, every particle finds the set of adjacent pixels it could potentially move to. It then computes the energy differences of all possible moves. Moves that lead to topological violations are pruned from the list. Then, a graph of causally dependent moves is constructed. An example of causal dependency is illustrated in Fig 2, where the possible moves of particle **p** depend on the move of particle **q**. Assume that the energetically most favorable move for particle **p** is downward (Fig 2a). If the energetically most favorable move of particle **q** is to go left (Fig 2b), this violates the topological constraint that the light-gray region has to be a 4-connected set of pixels (Fig 2c). Simply executing the energetically most favorable move for each particle could hence lead to topological violations. In the situation shown in Fig 2, only one of the two particles can execute its most favorable move, constraining the possible moves of the other. In the present greedy descent scheme, the move that leads to the largest energy decrease has priority.

**Fig 3:**
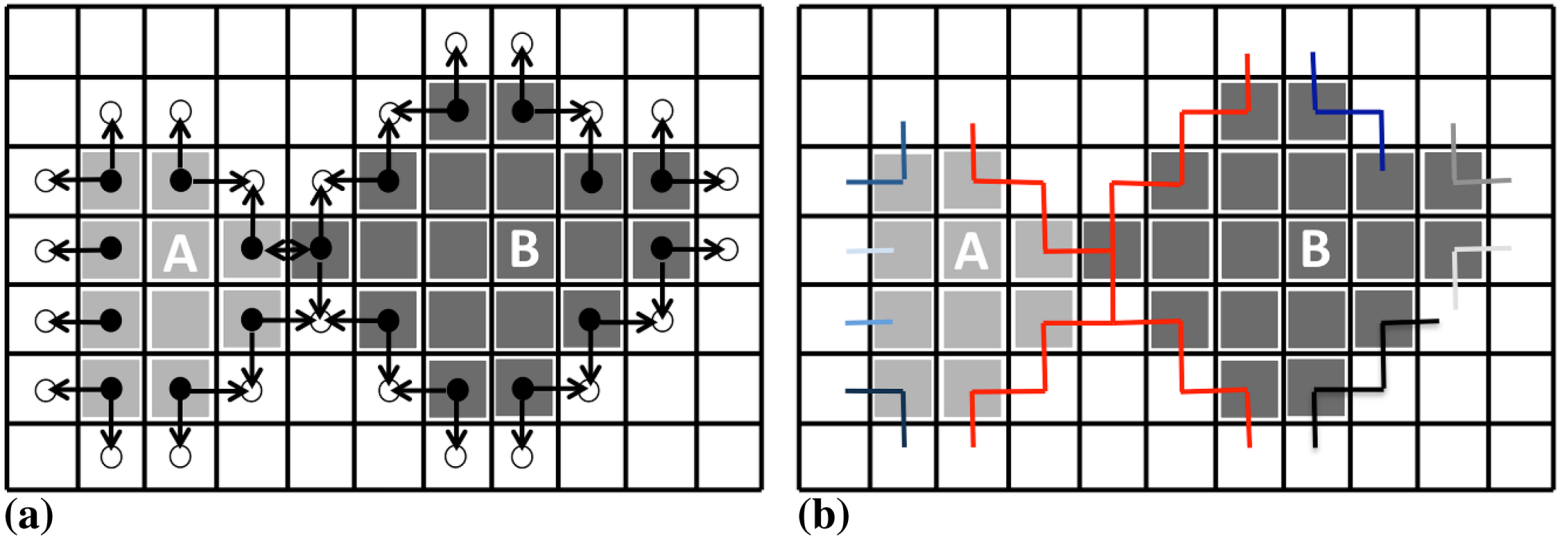
Illustration of the dependency graph construction. (a) All possible moves are enumerated for all particles. (b) The undirected graph of causal dependencies is obtained by removing directionality and joining edges that share a common pixel. The maximal connected sub-graphs are represented by different colors.

In order to find the set of moves that can be executed concurrently, we build a graph of all such causal dependencies and sort them by energy. Fig 3 illustrates the construction of this undirected graph of causal dependencies. It starts from enumerating all possible moves for all particles (Fig 3a). Shrinking a region is done by removing the respective particle and inserting new boundary particles. This is irrelevant for the dependency graph. The directionality of the moves is also irrelevant and is removed, yielding a set of undirected edges. A vertex is introduced wherever two edges meet in any pixel. This defines the final graph (Fig 3b). Moves that are connected by a path in the graph are causally dependent. Connected sub-graphs of the final graph (highlighted by different colors in Fig 3b) hence correspond to dependent sets of moves. They can extend across several particles, defining long-range chains of dependency.

Each maximal connected sub-graph can be processed independently. While the moves within a maximal connected sub-graph are causally dependent, there are no dependencies across different maximal connected sub-graphs. In order to find the energetically most favorable set of moves that can be executed simultaneously, the edges in each maximal connected sub-graph are sorted by energy difference. In each sub-graph, the move that leads to the largest decrease in energy is executed.

Splits and fusions of FG regions are topological changes that are allowed by the energy. They are detected using concepts from digital topology [15, 25, 27–29] and accepted if energetically favorable. The BG region is allowed to arbitrarily change its topology.

### Data distribution by domain decomposition

**Fig 4:**
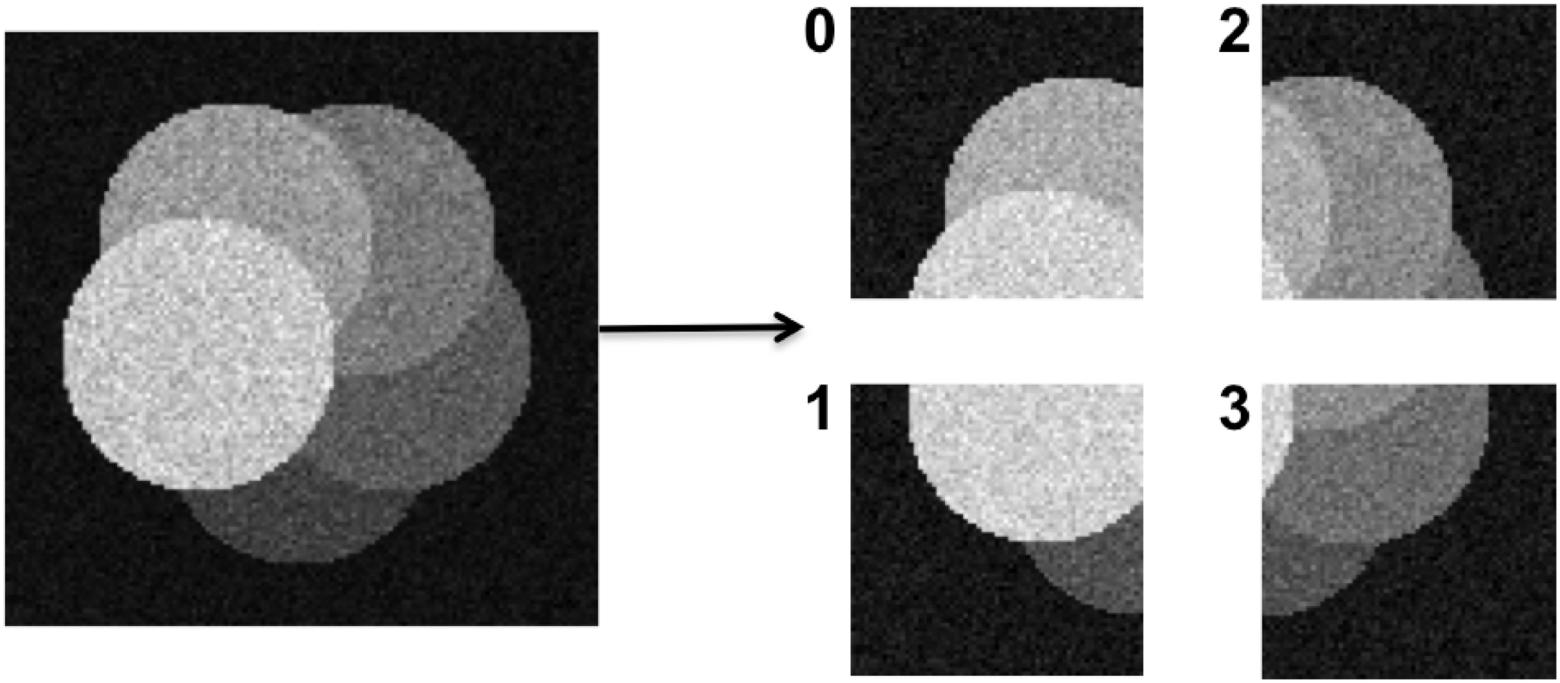
An illustrative example showing domain decomposition and distribution of an image across four computers (numbered 0 to 3).

We parallelize the DRC algorithm in a distributed environment by applying a domain-decomposition approach to the image. The input image is decomposed into disjoint sub-images that are distributed to different computers. This is illustrated in Fig 4. Domain decomposition and data distribution are done transparently by the PPM library [17–19]. Reading the input image from a file is also done in a distributed way, where each computer only reads certain image planes. The PPM library then automatically redistributes the data so as to achieve a good and balanced decomposition. Each computer only stores its local sub-image, and no computer needs to be able to store the entire image data.

**Fig 5:**
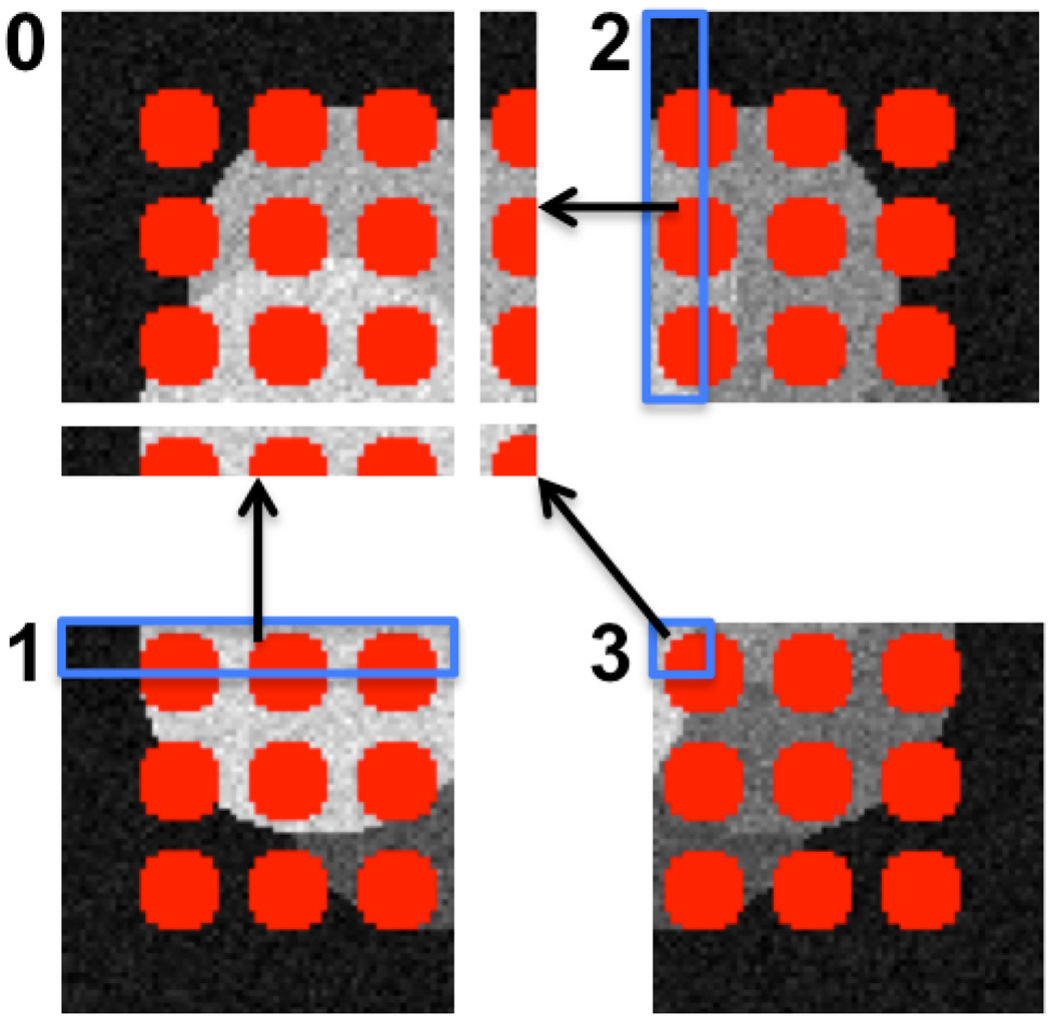
Ghost layers communicate information between neighboring sub-images residing on different computers. In the example from the previous figure, processor 0 receives ghost data from processors 1, 2, and 3, as shown for a ghost layer of 10 pixels width. The same is also done on all of the other processors. This allows the particles (boundary pixels of red regions) to smoothly migrate across computers, and energy differences to be evaluated purely locally on each sub-image.

The algorithm is then initialized locally on each computer, using only the local sub-image. The boundary information between sub-images is communicated between the respective computers with ghost layers. Ghost layers are extra layers of pixels around each sub-image that replicate data from the adjacent sub-images on the other processors, as illustrated in Fig 5. The width of these ghost layers is determined by the number of pixels required to compute energy differences, i.e., by the radius of the energy kernel (see Ref. [15] for details). The width of the ghost layer defines the communication overhead and hence the parallel scalability of the algorithm. PPM ghost mappings [17–19] are used to transparently update and manage ghost layer information whenever the corresponding pixels on the other computer have changed.

The initial segmentation from which the algorithm starts can be determined in a number of ways. Fig 5 shows an example of an initial segmentation given by uniformly distributed circles (shown in red). From there, the algorithm evolves to the final result. Using an initialization that is so far from the final result, however, increases the runtime and also bears the risk of getting stuck in a sub-optimal local energy minimum. In practice, we hence usually initialize by a local-maximum detection or an initial intensity thresholding.

**Fig 6:**
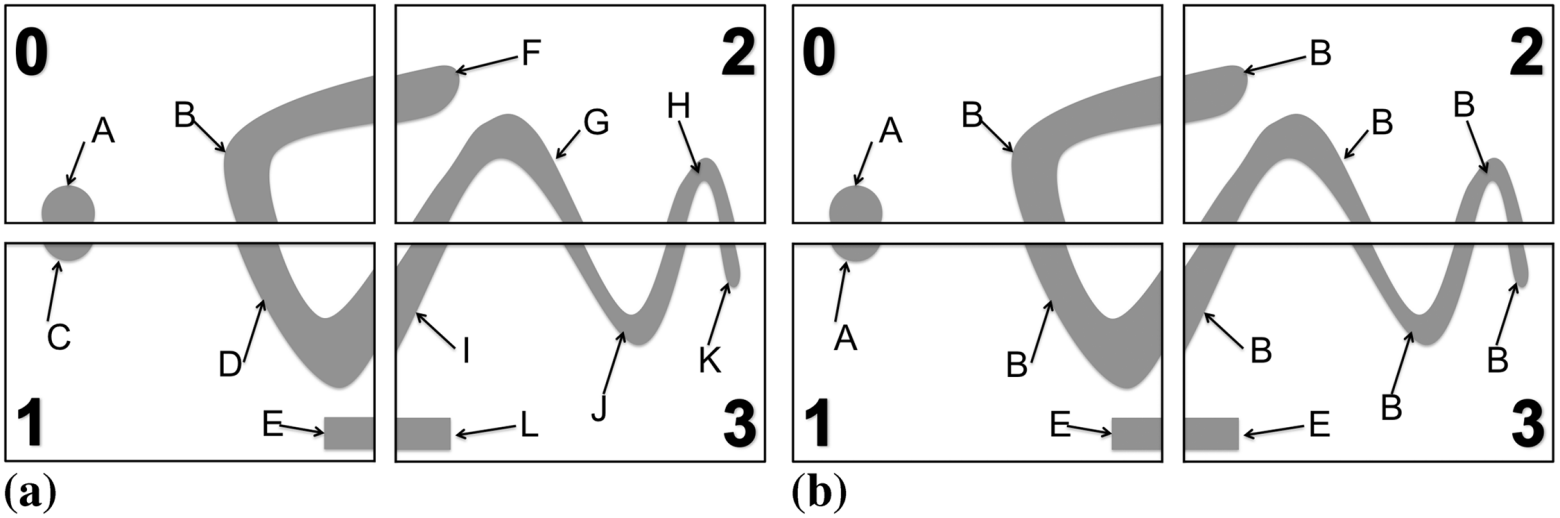
Region label initialization starts by each processor assigning unique labels to the FG regions in its sub-image (a). This, however, leads to conflicts for regions extending across multiple sub-images, as they will receive multiple, conflicting labels. Using a parallel connected-component algorithm [30], these conflicts are resolved in a second step, leading to a globally unique label for each FG region (b).

Starting from the initial segmentation, each FG region is identified by a globally unique label [15]. This requires care in a data-distributed setting, since different computers could use the same label to denote different regions. In our implementation, each processor first performs an intermediate local labeling of the regions in its sub-image. Using the processor number (i.e., processor ID), this is done such that no two labels are used twice (see Fig 6a). All regions are hence labeled uniquely. However, regions extending across more than one sub-image will be assigned multiple, conflicting labels. In a second step, the algorithm resolves these conflicts, ensuring that each FG region is uniquely labeled.

Using the definition that a FG region has to be a connected set of pixels, uniquely labeling them can be done using a parallel connected-component algorithm [30–35]. We here use the algorithm proposed by Flanigan *et al.* [30], which is based on an iterative relaxation process. During this, each sub-image exchanges boundary-crossing labels with neighboring processors. The labels are then replaced by the minimum of the two labels from the two processors. This process continues in iterations until a fixed point (labels do not change any more on any processor) is reached [30]. This is only done once, during initialization, and leads to a result as illustrated in Fig 6b. Every FG region now has a globally unique, unambiguous label, independent of which sub-image it lies in, or across how many computers the image has been distributed. This sets the basis for the energy-minimization iterations of the DRC algorithm.

### Parallel contour propagation

Following initialization and initial region labeling, particles move across the image as driven by the energy-minimization flow in order to compute the segmentation. As outlined in the section “Review of Discrete Region Competition”, this involves construction of a dependency graph of causally dependent particle moves, followed by selecting a maximal set of non-interfering moves. In a data-distributed setting, the problem occurs that every computer only knows the part of the graph that resides in its local sub-image. If graphs span across processor boundaries, correct move selection cannot be guaranteed without additional communication. This communication between computers is required to find a globally consistent set of independent moves, but should be kept to a minimum in order to guarantee algorithm scalability.

In our implementation, finding a globally consistent set of moves starts by each processor *P* creating a local undirected graph *G*_*P*_, comprising its interior and ghost particles. Disconnected parts of the graph that entirely lie within the local sub-image are called *interior sub-graphs* $G_{P}^{i}$. Parts of the graph that extend across sub-image boundaries are called *boundary sub-graphs* $G_{P}^{b}$.

Identifying compatible moves in an interior sub-graph can be done independently by each processor. Resolving boundary sub-graphs, however, requires information from all sub-images across which the sub-graph extends. This is challenging because the sub-graphs sizes, structures, and distributions are not known *a priori*, as they depend on the input image data.

**Fig 7:**
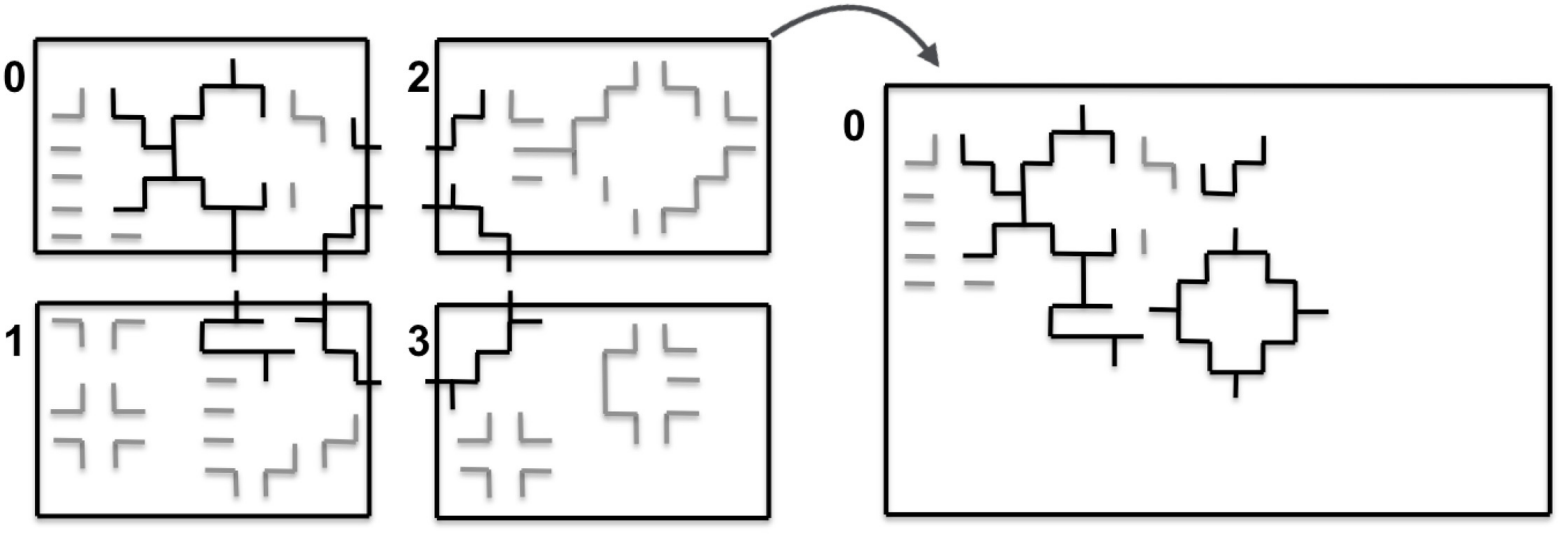
The master-slave approach to finding the global independent move set by gathering all boundary sub-graphs on a single master processor and then sending back the results. In this example, processor 0 is the master.

Traditionally, master-slave approaches have been used to solve this problem on distributed machines. This is illustrated in Fig 7. In this approach, the boundary sub-graphs $G_{P}^{b}$ from all processors are gathered on one single processor, the master processor. This master processor then determines the move sets and sends them back to the respective other processors. Meanwhile, the other processors work on their interior sub-graphs.

This approach is easy to implement, but carries substantial overhead due to the global communication and the task serialization on the master processor. As we show below, this approach does not scale and prevents acquisition-rate image analysis.

**Fig 8:**
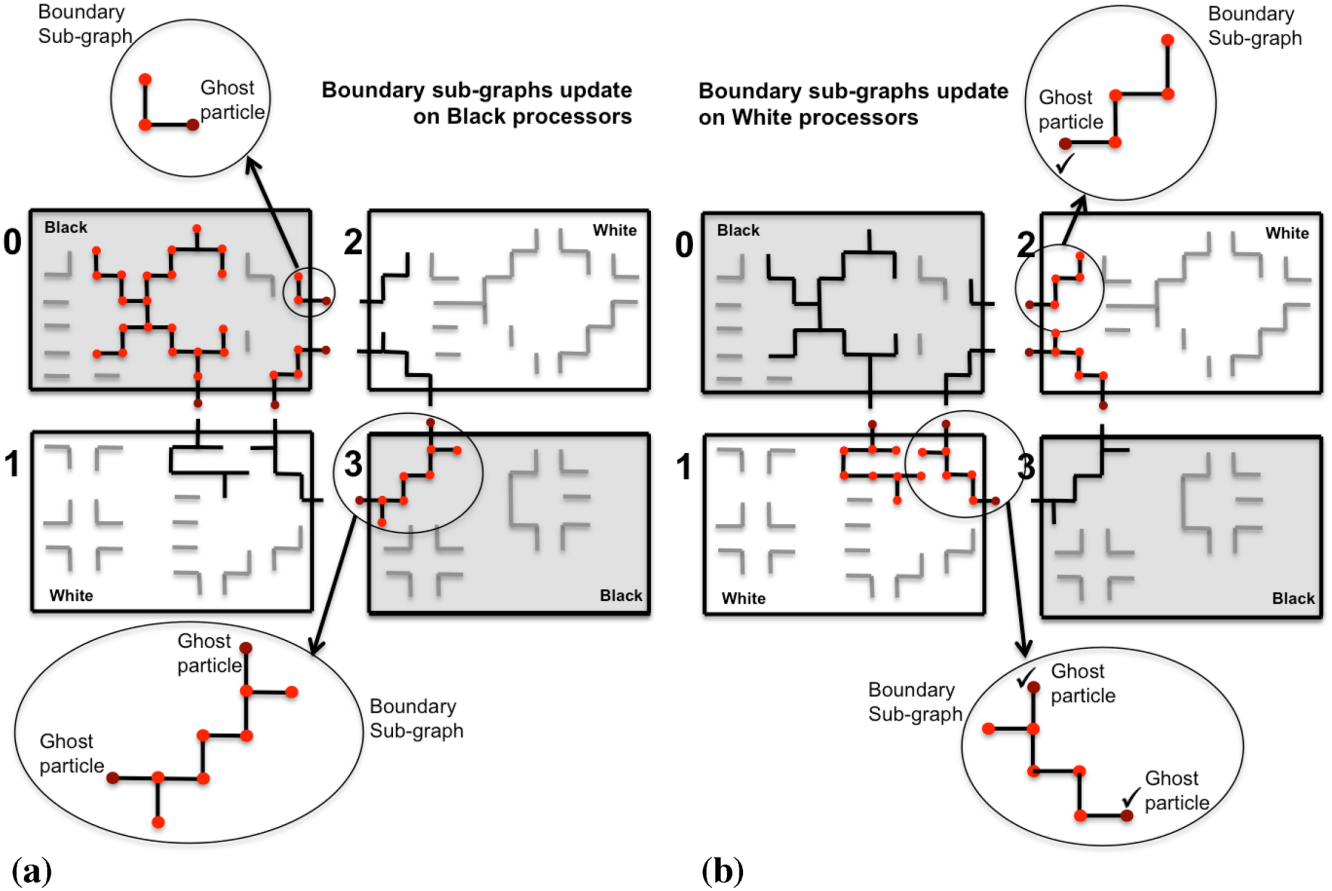
Distributed sub-graph algorithm to determine a globally consistent set of particle moves. The processors are divided into black and white ones using a checkerboard decomposition. (a) Compatible moves are identified simultaneously on all boundary sub-graphs (black) on the black processors, while the white processors work on their interior sub-graphs (gray). (b) Boundary particles (dark red) are send from the black to the white processors in order to provide the boundary condition for the boundary sub-graph processing on the white processors. The ghosts are not altered by the white processors, but immediately accepted as moves (symbolized by the check marks). This avoids conflicts and only requires local communication.

We address this problem by introducing a new parallel contour propagation algorithm, which does not require global communication and incurs no serialization. In theory, it hence scales perfectly. Instead of gathering all boundary sub-graphs $G_{P}^{b}$ on one master processor, we propose to use the locally available boundary sub-graph on each processor and identify the compatible moves only on that local part. If all processors did this in parallel, however, conflicting moves across sub-image boundaries would occur. We avoid this by decomposing the processors into two sets: black and white processors. Since FG regions are face-connected, using a checkerboard decomposition as illustrated in Fig 8 ensures that boundary sub-graphs always cross from black to white processors, or vice versa. They never cross sub-image boundaries within one color, hence avoiding boundary conflicts if the processing is done by color. Therefore, the black processors start by determining the viable moves on their boundary sub-graphs, while the white processors work on their interior sub-graphs. Then, the black processors communicate their boundary decisions to the neighboring white processors using a ghost particle mapping [17]. Finally, the white processors resolve their boundary sub-graphs using the received decisions as boundary conditions, while the black processors work on their interior sub-graphs. This procedure is illustrated in Fig 8. It effectively avoids conflicts and determines a globally viable move set within two rounds of local ghost communication.

Taking advantage of non-blocking MPI operations, the whole procedure is executed in an asynchronous parallel way, as detailed in Algorithm 1. This largely hides the communication time of the ghost mappings, resulting in better scalability and speed-up on a distributed memory parallel machine.

**Algorithm 1**: Parallel distributed-memory contour propagation algorithm

**Find**: interior and boundary maximal connected sub-graphs, $G_{P}^{i}$ and $G_{P}^{b}$

**if** *Black processor* **then**

 **Receive**: ghost information from white neighbor processors

 **foreach** *boundary sub-graph* $G_{P}^{b}$ **do**

  identify compatible moves

 **Send**: non-blocking send of updated boundary particle information to white neighbor processors

 **foreach** *interior sub-graphs* $G_{P}^{i}$ **do**

  identify compatible moves

 **Wait**: for non-blocking send to complete

**if** *White processor* **then**

 **Send**: non-blocking send of boundary particle information to black neighbor processors

 **Receive**: non-blocking receive of ghost information from black neighbor processors

 **foreach** *interior sub-graph* $G_{P}^{i}$ **do**

  identify compatible moves

 **Wait**: for non-blocking receive to complete

 **foreach** *boundary sub-graphs* $G_{P}^{b}$ **do**

  identify compatible moves under ghost constraints

Breaking the boundary sub-graphs along sub-image boundaries changes the sorting order of compatible moves, and hence the convergence trace of the algorithm in energy space. Enforcing boundary conditions at the break points of the boundary sub-graphs amounts to an approximation of the original problem. This approximation is not guaranteed to determine the same global move set as the sequential approach, because the moves are only sorted by energy locally in each sub-graph, and not globally in the entire graph. However, as long as the statistical distribution of break points in the graph is unbiased, the optimizer is still guaranteed to converge, albeit the path of convergence may differ. This is a famous result from Monte Carlo (MC) approaches to the Ising model [36], where it has been shown that unbiased randomization of the moves may even accelerate convergence toward an energy minimum. In our case, the distribution of break points is indeed unbiased. This is because it is the result of a domain decomposition that depends on the number of processors used, and the graphs depend on the unpredictable image content. Therefore, independent unbiased breaking is satisfied, and the distributed approach converges.

**Fig 9:**
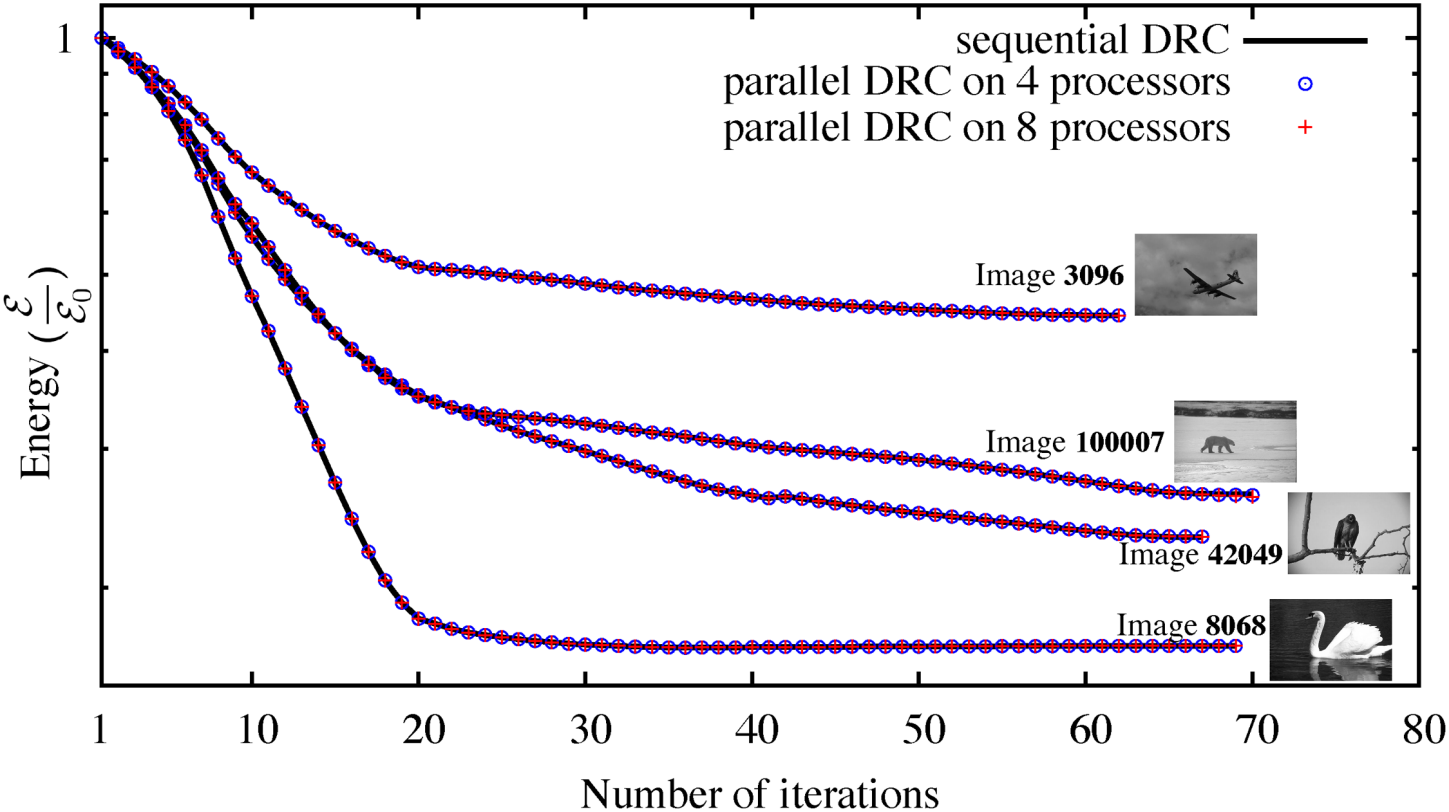
Energy evolution of the sequential DRC algorithm [15] and the present parallel algorithm on four different images from the Berkeley database [37] on 4 and 8 processors. Despite the boundary sub-graph decomposition (see main text), the results are pixel-wise identical in all cases except for image 100007, where two pixels on 4 processors and 3 pixels on 8 processors differ from the sequential result due to contour oscillations, as discussed in the main text.

We empirically confirm this convergence by comparing the energy evolution of the original sequential algorithm [15] and our new distributed method on different 2D benchmark images from the Berkeley database [37]. The result for four example images is shown in Fig 9 using different numbers of processors and hence different sub-graph decompositions. In all tested cases, both methods converge. The largest observed difference in final energy is less than 0.5%.

**Fig 10:**
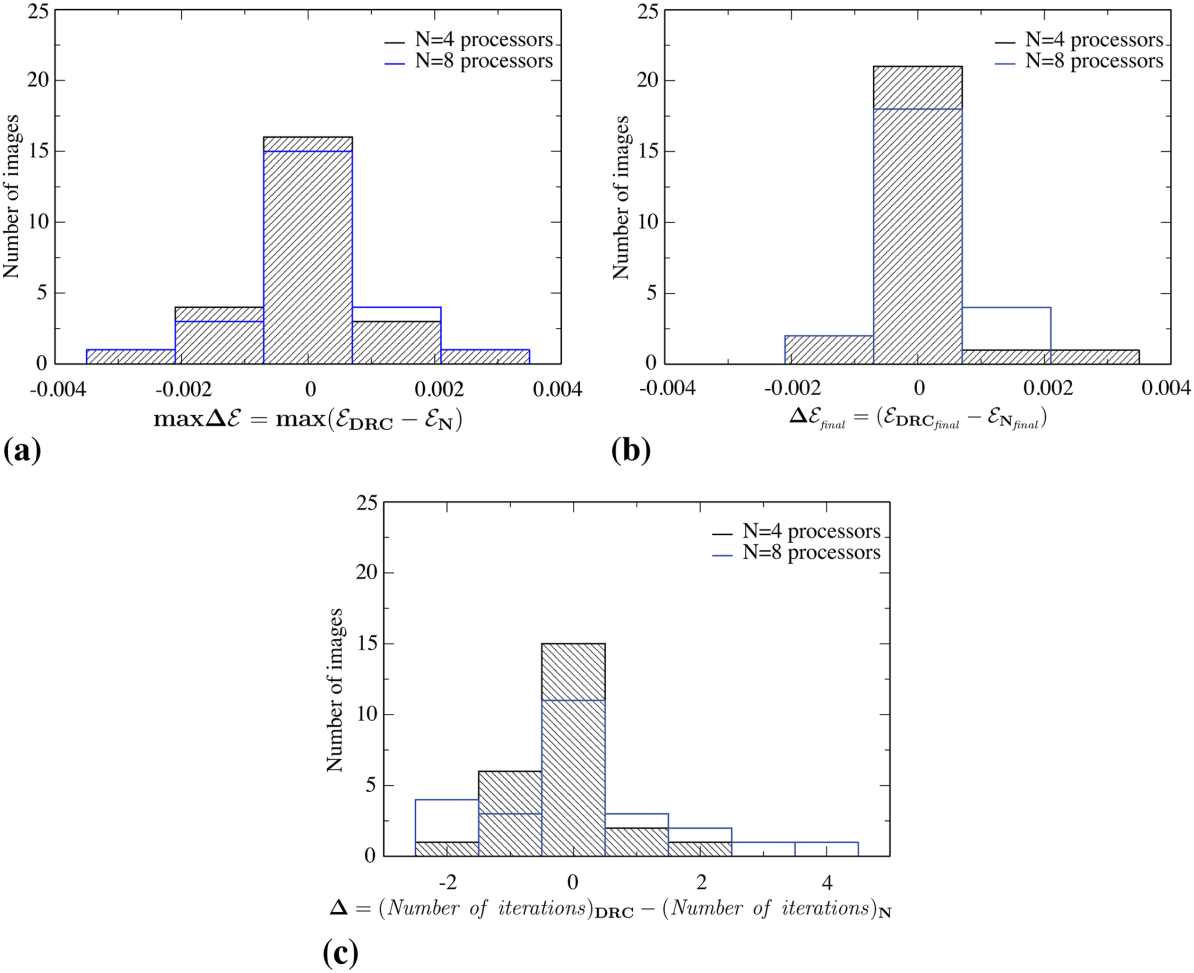
Comparison of the results from the distributed DRC algorithm and the original sequential implementation [15] on 25 2D images from the Berkeley benchmark database [37]. (a) Histogram of the maximum energy difference occurring anywhere along the energy evolution path; $E_{\text{DRC}}$ is the sequential algorithm and $E_{N}$ the distributed algorithm on *N* computers. (b) Histogram of the final energy difference of the converged solutions. (c) Histogram of the difference in the total number of iterations required to reach the converged final solution.

Fig 10 shows histograms of the energy differences for 25 images from the Berkeley database. Three metrics are shown: (a) the maximum energy difference occurring anywhere along the convergence path, (b) the difference in the energy of the final converged state, (c) the difference in the number of iterations requires to reach convergence.

The results in Figs 9 and 10 show that the parallel algorithm is in good agreement with the original sequential algorithm [15]. Both algorithms show the same energy-evolution trend and converge to almost the same energy level with less than 0.5% difference anywhere during energy evolution. Fig 10c also confirms the Ising-model result that the randomized parallel algorithm on average converges in fewer iterations than the sequential method.

**Fig 11:**
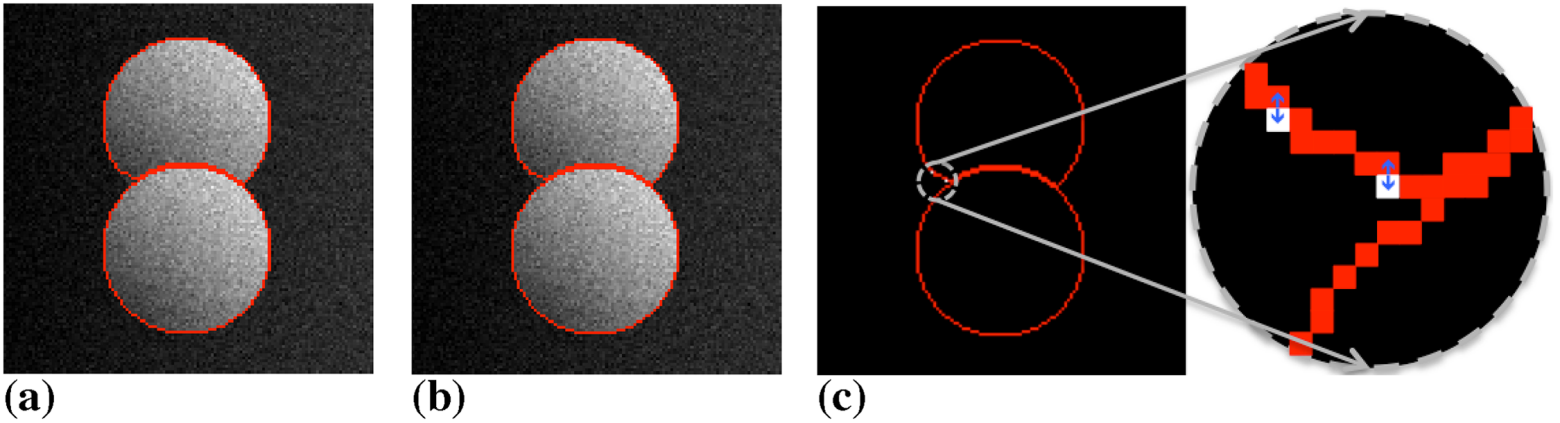
The small energy differences between the distributed and the sequential DRC implementations result from local pixel oscillations. An example is shown with a synthetic image using a piecewise smooth image model for segmentation. (a) Result on a single computer. (b) Result from the distributed algorithm on 8 computers. (c) Overlay of the two results with differences shows in white. These are two oscillatory particles jumping back and forth between two neighboring pixels. The final segmentation results are hence very close and amount to alternative pixelations of the object border line.

The question arises, however, if these small energy differences are significant in terms of the final segmentation. While no general guarantee can be given, the final segmentations were close in all cases tested, with at most 3 pixels differing between the sequential and parallel solutions. All observed pixel differences stemmed from contour oscillations around the converged state, as confirmed by pixel-wise comparison of the final segmentations. These oscillations are an inherent property of the energy descent method used in DRC [15]. They are suppressed by reducing the number of concurrently accepted moves whenever oscillations occur [15]. This is required in order to guarantee convergence of DRC. In our distributed DRC implementation, oscillations are detected locally by each computer. Also, the number of accepted moves per iteration is set locally, and potentially differently, by each computer. The oscillation pattern close to the final converged state is hence different than the one in sequential DRC. An example is shown in Fig 11 where the only differences between the two segmentations are the two oscillatory particles shown in white. Since they may stop their oscillations at different locations, the final segmentations may differ in these two pixels, which explains the small energy difference. The final segmentations are, however, geometrically close, and the algorithm converges toward the same local energy minimum. It is also important to keep in mind that the sequential DRC algorithm uses an approximate local optimizer that may not find the globally best segmentation. Sometimes, the slightly different result obtained by the distributed method is therefore better in terms of energy (see Fig 10b).

### Parallel topology processing and data-structure update

After having determined the set of compatible acceptable moves, the particles (and hence the contours) propagate in parallel on each processor. This changes the region labels of the corresponding pixels, as regions move, shrink, or grow. Particles that move across sub-image boundaries are communicated to the respective destination processor using the local neighborhood mappings of PPM [17]. This ensures global consensus about the propagating contours.

**Fig 12:**
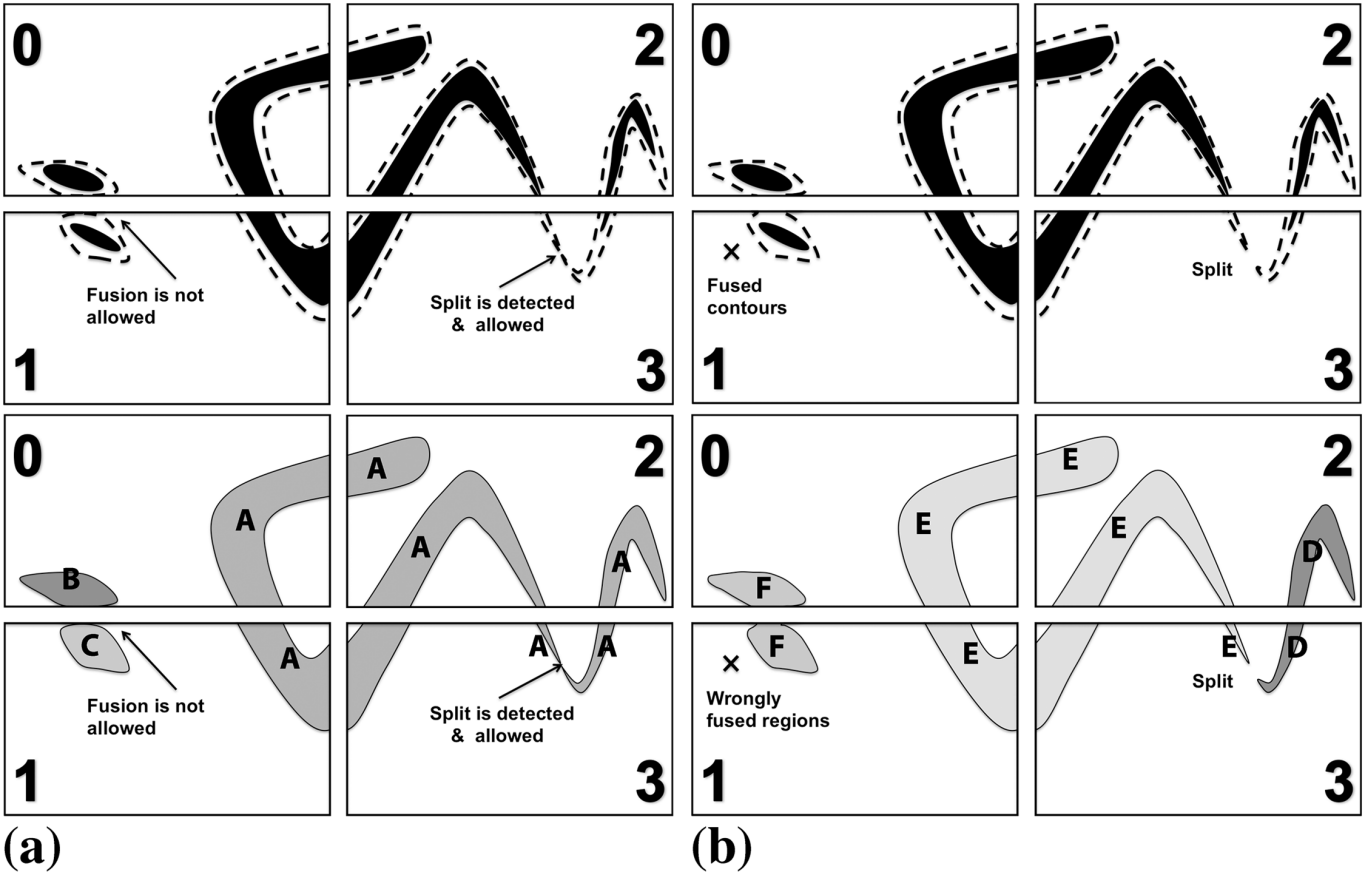
Distributed region split and merge algorithm. In the upper row, the evolving contours are shown by dashed lines and the underlying objects to be segmented by the black solid regions. (a) The situation before re-labeling the regions. Two regions (B/C) touch at a sub-image boundary, but should not fuse according to the image energy model. The region A extends across multiple sub-images and splits in sub-image 3. (b) Applying a parallel connected-component algorithm [30] would erroneously fuse regions touching at sub-image boundaries and unnecessarily re-label all regions with new unique labels.

In addition to propagating, contours can also split or fuse if that is energetically favorable. This corresponds to a region splitting into two, or two regions merging. While digital topology allows such topological changes in the segmentation to be efficiently detected using only local information [15], the labels of the involved regions may change across sub-image boundaries. Whenever region labels change as a result of a split or fusion, a seeded flood-fill is performed in the original DRC algorithm [15], in order to identify the new connected components. This again requires additional care in a distributed setting, as illustrated in Fig 12.

Fig 12a shows the two critical situations: two regions touching at a sub-image boundary that are not supposed to fuse (by the image energy model) and a split in a region that extends across multiple sub-images. The parallel connected component algorithm [30] used during initialization would unnecessarily re-label all regions that cross any sub-image boundary and erroneously fuse the two touching regions (Fig 12b). In order to obtain the correct result, we propose a particle-based alternative, as detailed in Algorithm 2.

**Algorithm 2**: Distributed region re-labeling

**Reconstruct**: label image *L*

*hotpart* ← *false*

*Create an empty list* *S*

**if** *contour particle label changes at sub-image boundary* **then**

 *activate particle as a hot particle*

 *hotpart* ← *true*

**Ghost mappings**: Particle

**if** *there is any hot ghost particle* **then**

 add it as a seed to the list *S*

**Global**: reduce operation on *hotpart*

**while** *hotpart* **do**

 Reconstruct label image *L* using flood fill from the seeds in *S*

 **Empty**: *S*

 **Ghost mappings**: Particle

 **if** *there is any hot ghost particle* **then**

  add it as a seed to the list *S*

 *hotpart* ← *false*

 **if** *contour particle label changes at particle ghost layer* **then**

  *activate particle as a hot particle*

  *hotpart* ← *true*

 **Global**: reduce operation on hotpart

**Fig 13:**
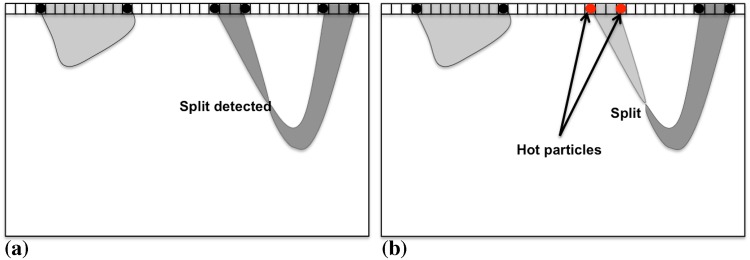
Boundary particles are activated upon region label changes in the local sub-image. Only activated (“hot”) boundary particles are communicated to the neighboring processor, restricting re-labeling to affected regions and avoiding communication of a full ghost layer of pixels. (a) All boundary particles (black disks) are deactivated (“cold”) before local region label update. (b) Boundary particles of re-labeled regions are activated (red disks, “hot”) and propagate the label change to the neighboring processor.

Taking advantage of the particle representation of the evolving contours, information about region label changes at sub-image boundaries is communicated through PPM’s ghost-get mappings [17]. For each region, however, only two particles are communicated instead of the full ghost layer of pixels. This is illustrated in Fig 13. Moreover, this only happens when the region label on the source processor did actually change. In this case, the corresponding boundary particles are activated. By default, all boundary particles are deactivated. Activated particles, which we call “hot particles” from now on, as inspired by the classical forest-fire algorithm, are then sent to the neighboring processor.

The neighboring processor receiving the hot ghost particles starts a local forest-fire algorithm for seeded flood filling of the region, using the hot ghosts as seeds. Since this may propagate the label change across multiple processors, the procedure proceeds in iterations until no more hot particles are detected anywhere. This is determined by a global all-reduce operation of local flags for the presence of hot particles in each sub-image. Regions are always re-labeled using the lower of the two labels. This means that hot particles only propagate changes with new labels lower than existing ones. Therefore, the procedure is guaranteed to terminate, as oscillations or loops cannot occur.

The complete procedure is detailed in Algorithm 2. Again, all communication (mappings) is done asynchronously using non-blocking MPI operations. After execution of the algorithm, all regions are again identified by globally unique labels, but only necessary changes are made. Regions that did not undergo topological changes retain their previous labels. This prevents spurious region fusions and keeps the data-structure updates to a minimum. Also, communicating only hot ghost particles, instead of full pixel layers, significantly reduces the communication overhead.

## Results

We first show correctness and efficiency of the distributed parallel algorithm and then illustrate its application to acquisition-rate image segmentation in 3D light-sheet fluorescence microscopy. We demonstrate correctness by comparison with the original reference implementation of Cardinale *et al.* [15] on synthetic and real-world images taken from the original DRC paper [15]. Then, we assess the parallel efficiency and scalability of the present implementation using scalable synthetic images in both a weak-scaling and a strong-scaling experiment.

All computations were performed using the PPM library [17–19] in its 2015 version on the Bull cluster “taurus” at the Center for Information Services and High Performance Computing (ZIH) of TU Dresden. The cluster island used consists of 612 Intel Haswell nodes with 24 cores per node and 2.5 GB of main memory per core. The parameter settings for all test cases are summarized in Table 1. They were determined following the guidelines given in the original DRC publication [15].

**Table 1:** Parameter settings used for the cases shown in this paper (PC: piecewise constant; PS: piecewise smooth). See Ref. [15] for parameter meaning and guidelines.

Initialization	Algorithm parameters	$E_{\text{data}}$	Energy parameters
Icecream PC 2D, 130 × 130, Fig 14			
6 × 6 bubbles	*θ* = 0.02, *R*_*κ*_ = 4	PC	*λ* = 0.04
Bird, 481 × 32, Fig 16			
32 × 21 bubbles	*θ* = 4.5, *R*_*κ*_ = 5	PC	*λ* = 0.2
Cell nuclei, 672 × 512, Fig 17			
local maxima	*θ* = 0.02, *R*_*κ*_ = 4	PC	*λ* = 0.06
Icecream PS 2D, 130 × 130, Fig 18			
5 × 5 bubbles	*θ* = 0.2, *R*_*κ*_ = 4	PS	*λ* = 0.04, *β* = 0.05, *R* = 8
Elephants 2D, 481 × 321, Fig 20			
21 × 14 bubbles	*θ* = 0.2, *R*_*κ*_ = 8	PS	*λ* = 0.2, *β* = 0.05, *R* = 4
Zebrafish embryo germ cells 3D, 188 × 165 × 30, Fig 21			
bounding box	*R*_*κ*_ = 4	PS	*λ* = 0.08, *β* = 0.005, *R* = 9*μ*m
Synthetic unit cell test image 3D, 256 × 256 × 256, Fig 22			
local maxima	*θ* = 0.02, *R*_*κ*_ = 4	PC	*λ* = 0.04
Drosophila embryo 3D, 1824 × 834 × 809, Fig 25			
local maxima from blob detector	*θ* = 0.001, *R*_*κ*_ = 8	PC	*λ* = 0.005
Drosophila embryo 3D, 1824 × 834 × 809, Fig 26			
local maxima from blob detector	*θ* = 0.001, *R*_*κ*_ = 8	PS	*λ* = 0.005, *β* = 1.0, *R* = 8
zebrafish vasculature 3D, 1626 × 988 × 219, Fig 27			
thresholding	*θ* = 10.0, *R*_*κ*_ = 8	PS	*λ* = 0.02, *β* = 0.001, *R* = 12

### Correctness of the distributed algorithm

#### Results using a piecewise constant image model

**Fig 14:**
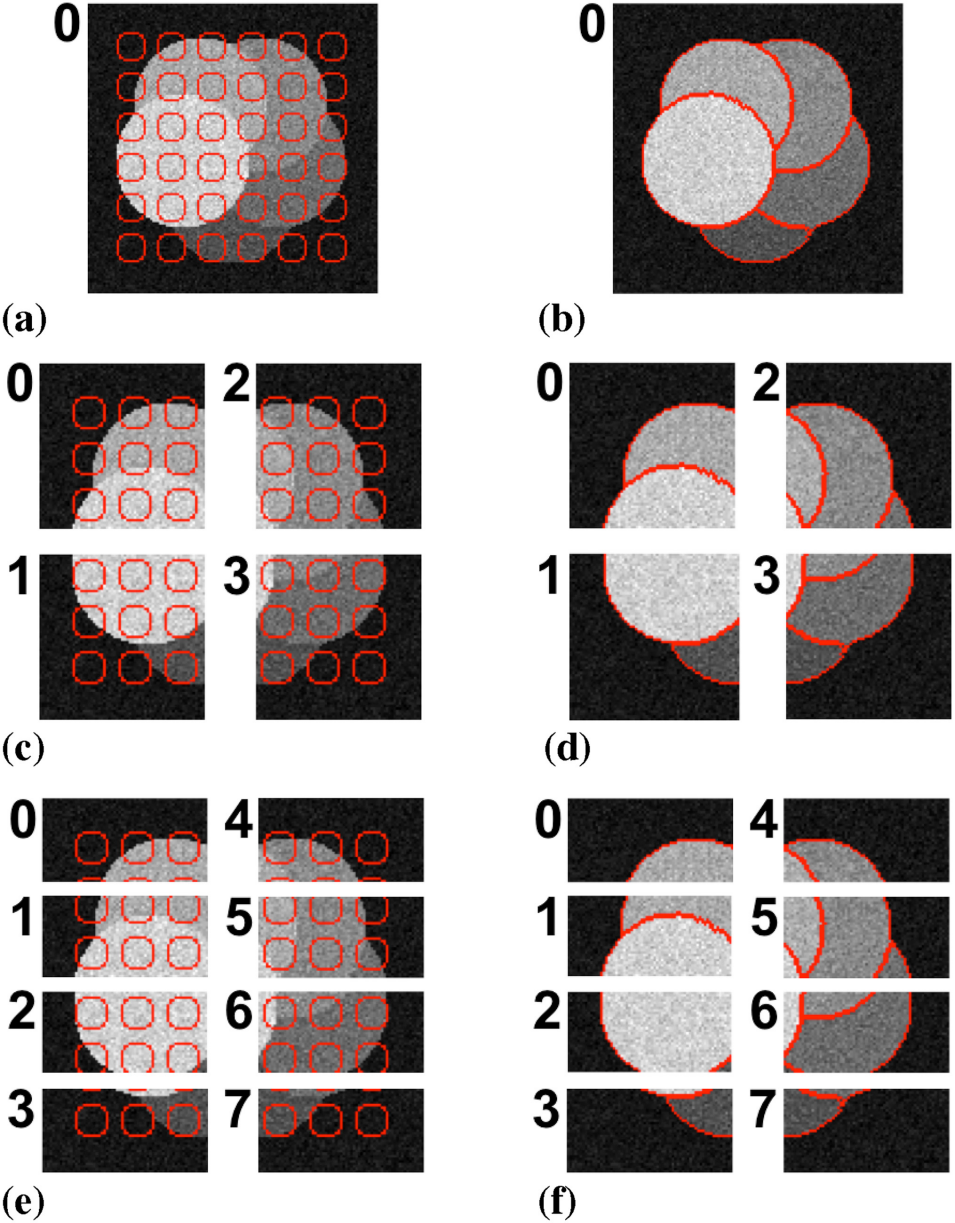
Distributed segmentation of a synthetic test image using a piecewise constant image model. (a) Initialization on a single processor with particles shown in red. (b) Final result on a single processor. (c) Initialization on four processors. (d) Final result on four processors. (e) Initialization on eight processors. (f) Final result on eight processors. The results are identical to those in Ref. [15] in all cases.

We first check that the distributed algorithm produces the same results as the sequential benchmark implementation in the case of a multi-region piecewise constant (PC) image model. In this model, the assumption is that different FG regions have different intensities that are, however, spatially constant within each region. We use the same test image as in Ref. [15] in order to compare the results. The result from the present distributed implementation is shown in Fig 14 using one, four, and eight processors. Pixel-wise comparison shows that all segmentation results are identical to the one reported in Ref. [15] for the sequential benchmark implementation.

**Fig 15:**
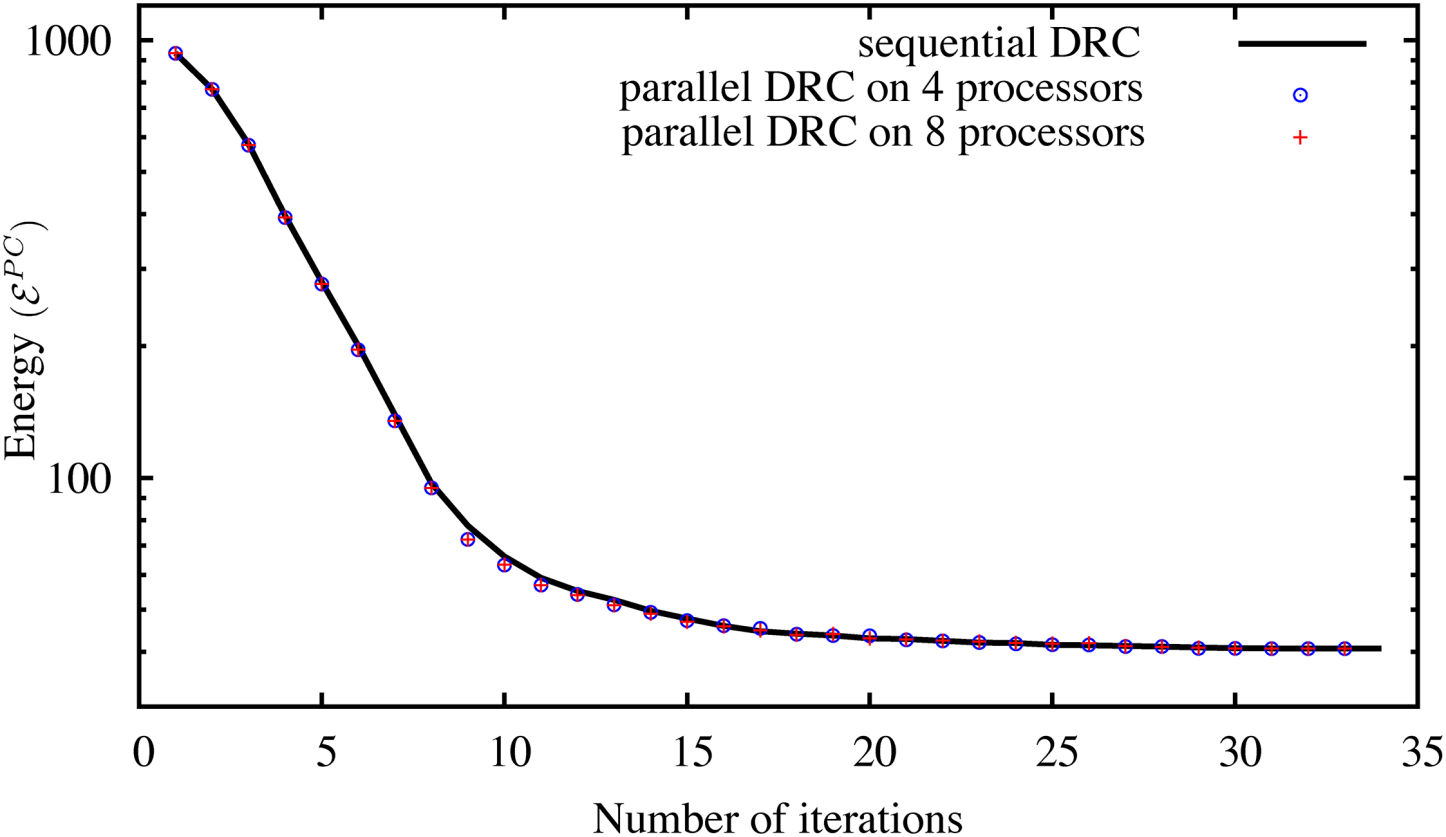
Energy evolution of the sequential DRC algorithm of Cardinale *et al.* [15] in comparison with the present distributed algorithm processing the piecewise constant test image from Fig 14 on four and eight processors.

Fig 15 shows the energy evolution of the distributed cases compared with the original sequential benchmark implementation of Cardinale *et al.* [15]. If anything, the graph randomization used in the present distributed algorithm slightly accelerates convergence in the first half of the iterations. All methods reach the same final energy.

**Fig 16:**
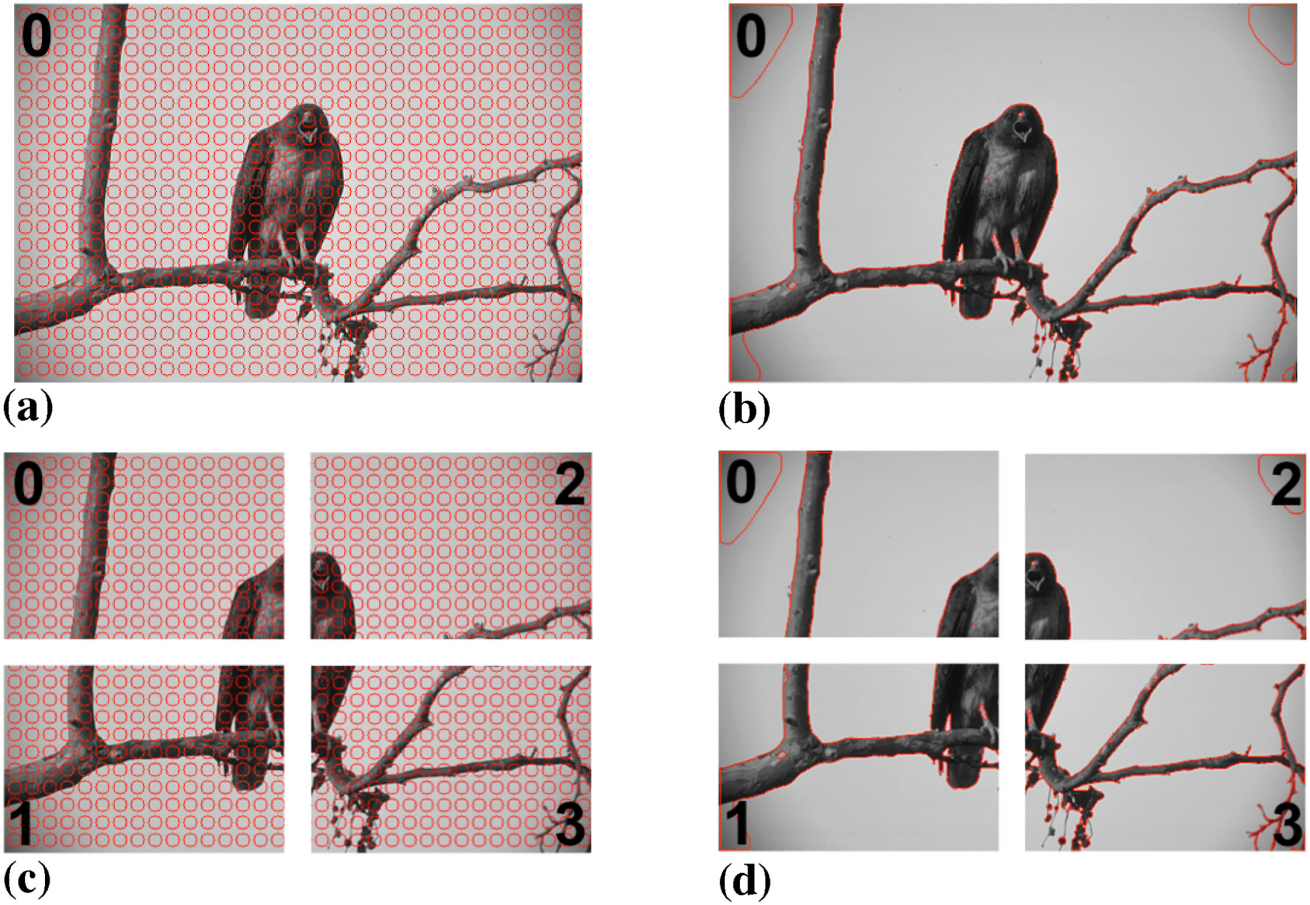
Distributed segmentation of a natural-scene image using a piecewise constant image model. (a) Initialization on a single processor with particles shown in red. (b) Final result on a single processor. (c) Initialization on four processors. (d) Final result on four processors. The results are identical to those in Ref. [15] in both cases.

We further compare the results from the present distributed implementation with the original sequential algorithm on real image data. The same natural-scene image as considered in the original publication [15] is shown in Fig 16. Again, the present implementation running on one and four processors produces the exact same result as the benchmark implementation.

**Fig 17:**
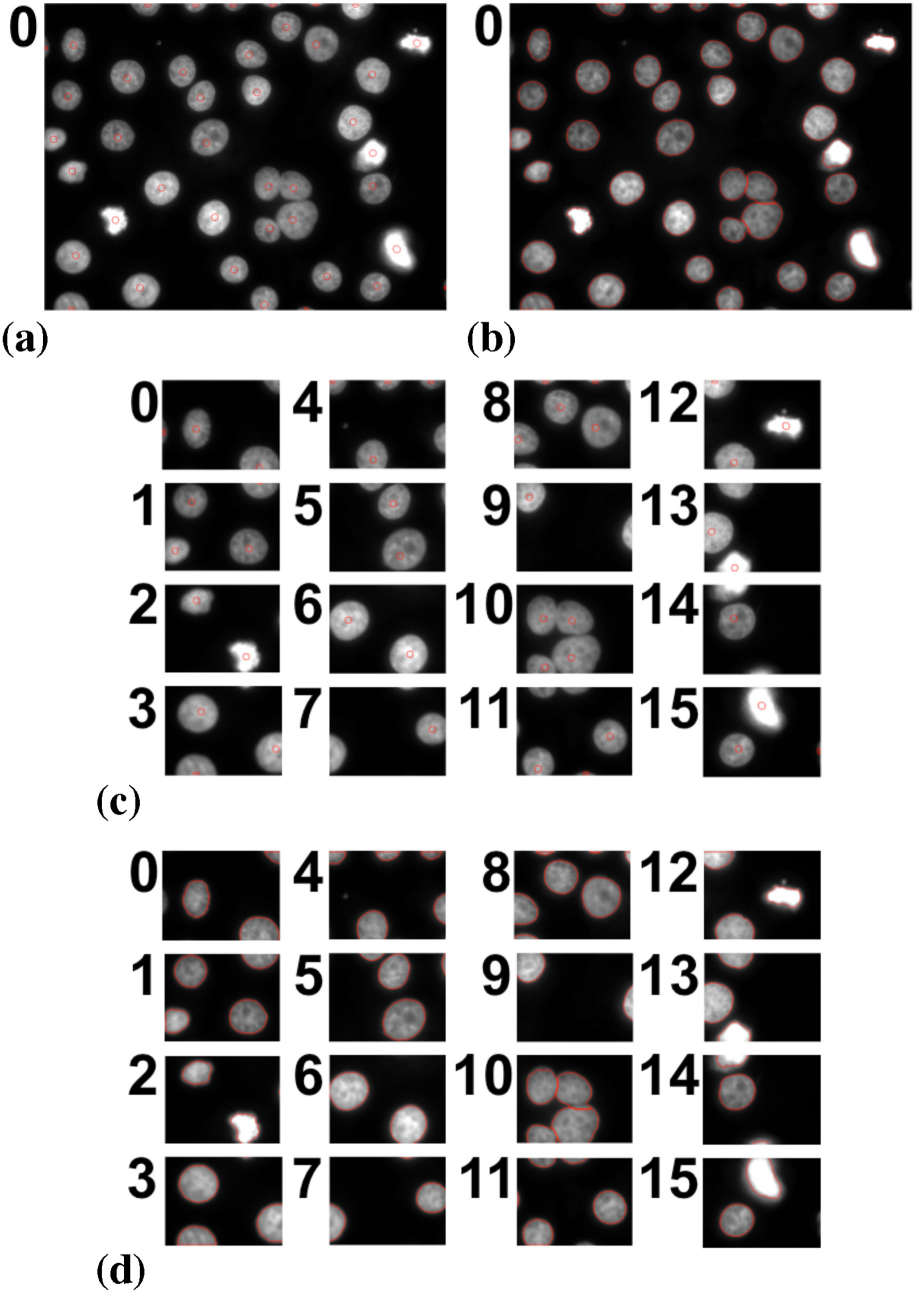
Distributed segmentation of fluorescently labeled cell nuclei (raw image: Dr. Prisca Liberali, FMI Basel) using a piecewise constant image model. (a) Initialization on a single processor. (b) Result on a single processor. (c) Initialization on 16 processors. (d) Result on 16 processors. The results are identical to those in Ref. [15] in both cases.

As a second real image, we consider the same fluorescence microscopy image of nuclei as in the original publication [15]. The results on one and 16 processors are shown in Fig 17. The algorithm is initialized with a circular region around each local intensity maximum after blurring the image with a Gaussian filter of width *σ* = 10 pixel. This is the same initialization as used in Ref. [15]. The results are identical, pixel by pixel.

#### Results using a piecewise smooth image model

**Fig 18:**
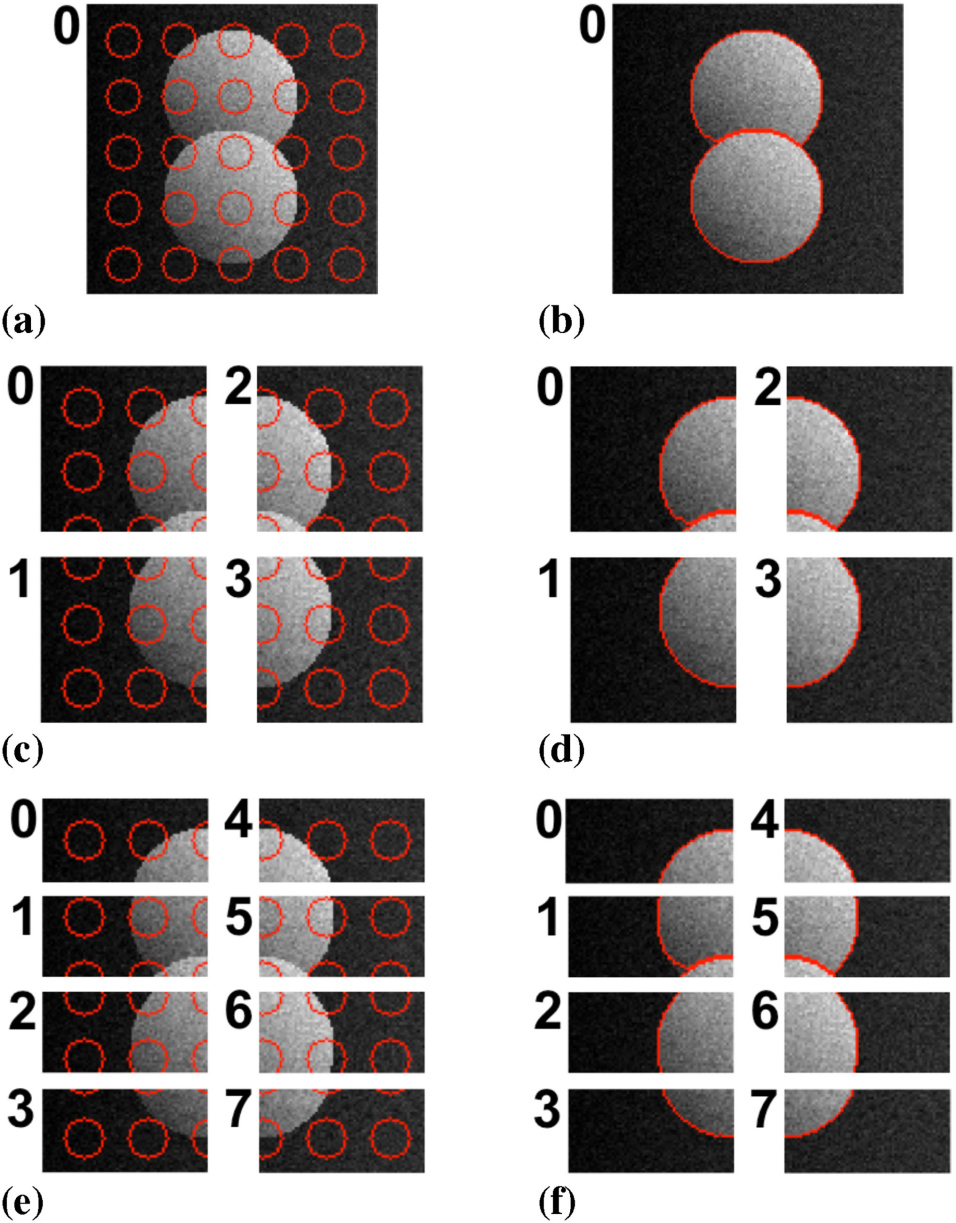
Parallel segmentation of a synthetic image using a piecewise smooth image model. (a) Initialization on a single processor with particles shown in red. (b) Final result on a single processor. (c) Initialization on four processors. (d) Final result on four processors. (e) Initialization on eight processors. (f) Final result on eight processors. Two oscillatory pixels differ with respect to the result in Ref. [15] (see also Fig 11).

The DRC algorithm is generic over a wide range of image models, including the more complex piecewise smooth (PS) model. In this model, each region is allowed to have a smooth internal intensity shading. We again use the same synthetic test image as in Ref. [15] and illustrate the result in Fig 18. Pixel-to-pixel comparison of the final segmentation results shows differences in two oscillatory pixels on eight processors (see also Fig 11). This is consistent with the way boundary oscillations are detected and handled in the distributed algorithm in comparison with the sequential one.

**Fig 19:**
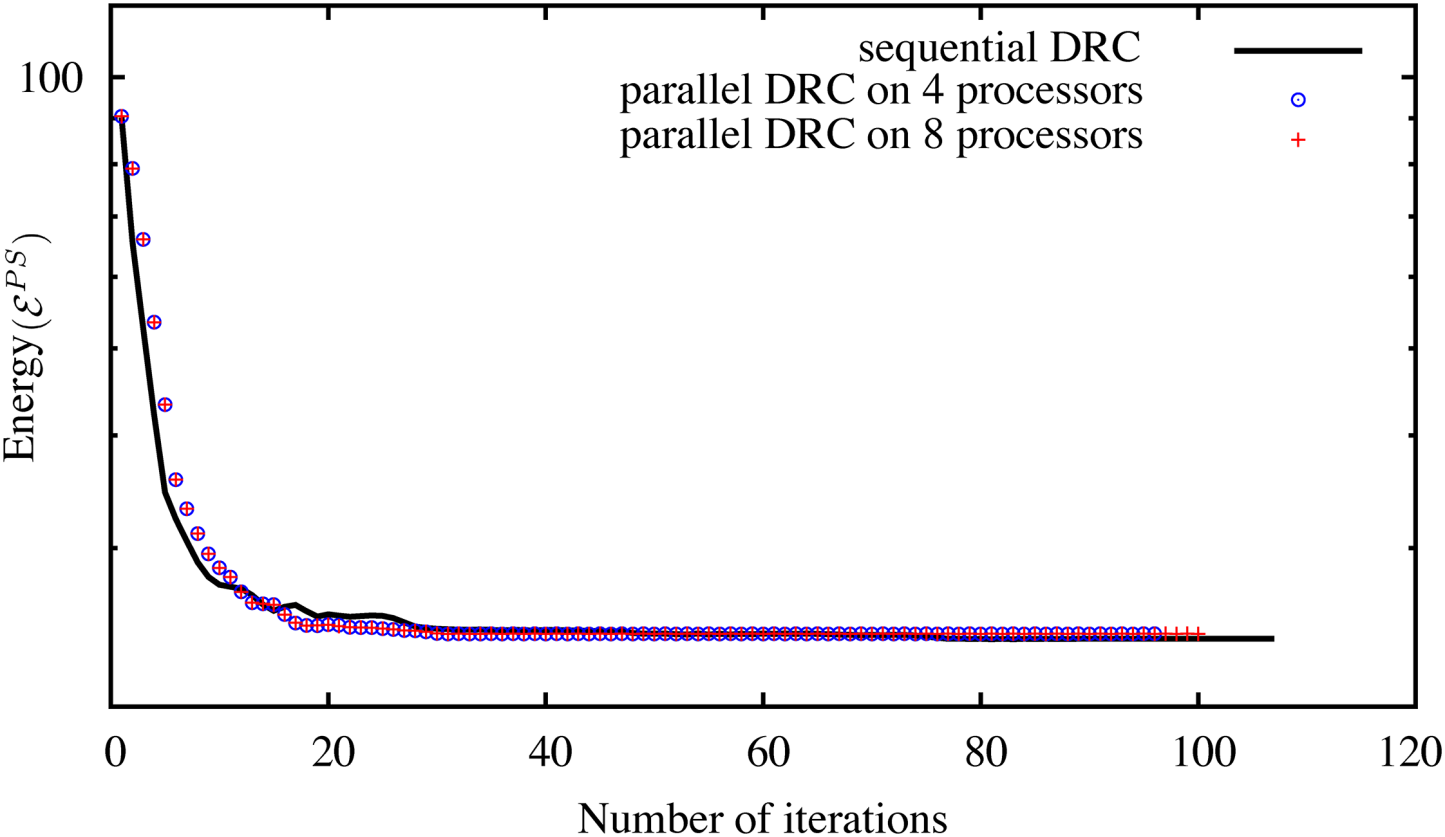
Energy evolution of the sequential DRC algorithm of Cardinale *et al.* [15] in comparison with the present distributed algorithm processing the piecewise smooth test image from Fig 18 on four and eight processors.

The energy evolution for this case is shown in Fig 19 in comparison with the original sequential DRC algorithm of Cardinale *et al.* [15]. Again, the two convergence traces are almost identical with small differences stemming from the graph decomposition used in the present implementation. The difference in final energy is due to the two oscillatory pixels, as discussed above and shown in Fig 11.

**Fig 20:**
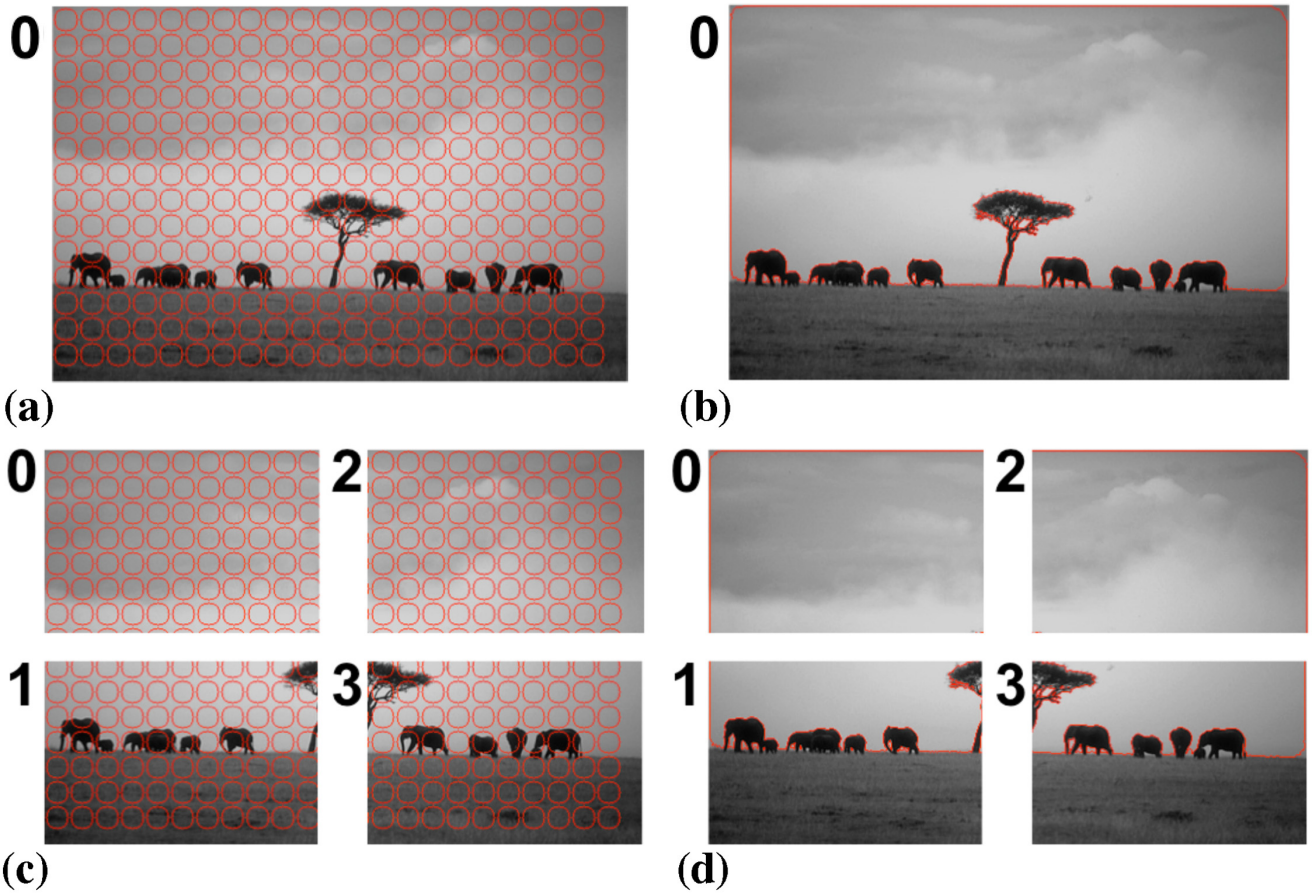
Distributed segmentation of a natural-scene image using a piecewise smooth image model. (a) Initialization on a single processor with particles shown in red. (b) Final result on a single processor. (c) Initialization on four processors. (d) Final result on four processors. The two results are identical.

Fig 20 illustrates the sequential and distributed segmentations of a natural-scene image using the PS image model. By pixel-to-pixel comparison, the segmentation result on four processors (Fig 20d) is identical to the one computed by a single computer (Fig 20b).

**Fig 21:**
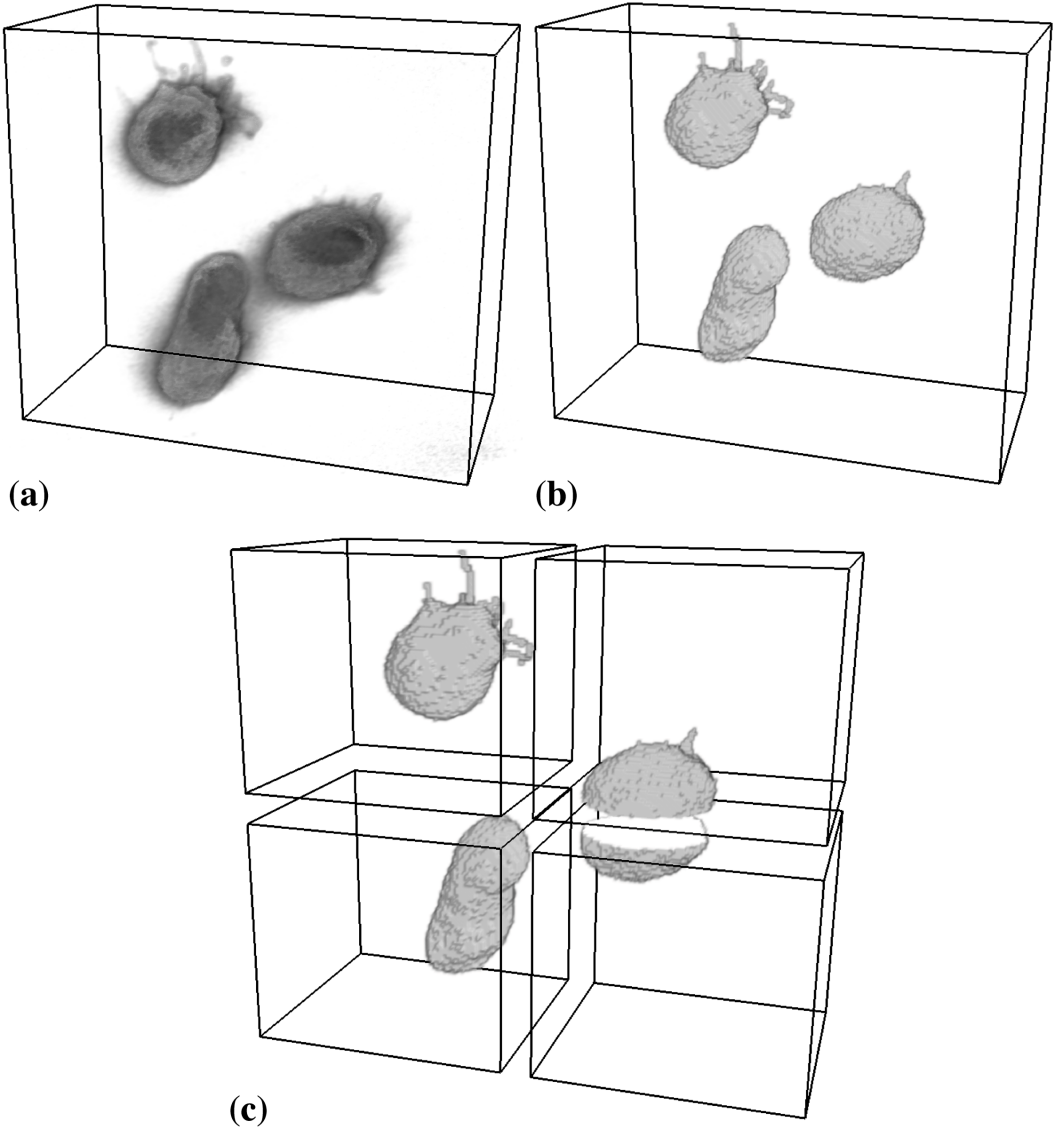
Distributed segmentation of zebrafish primordial germ cells using a piecewise smooth image model. (a) Raw 3D confocal fluorescence microscopy image showing three cells with a fluorescent membrane stain (image: M. Goudarzi, University of Münster). (b) Segmentation result on a single processor. (c) Segmentation result on four processors. The two results are identical.

As a first 3D test image, we use the same fluorescence confocal image of zebrafish germ cells that was also used in Ref. [15]. Fig 21 shows the raw image along with the PS segmentation results on one and four processors. By pixel-wise comparison, the results are identical.

### Efficiency of the distributed algorithm

Performance of a distributed parallel algorithm is influenced by many factors, including the structure of the input data, computer memory architecture, communication network, disk space, and background load. While it is impossible to reproduce or control all of these, we present empirical tests in order to assess the overall performance of the present algorithm in terms of parallel scalability and speed. Scalability (parallel efficiency) quantifies how well a distributed algorithm utilizes the computer resources as the number of computers/processors increases. Therefore, we provide results for both weak and strong scaling on synthetic benchmark images. Weak scaling measures how well the algorithm scales to very large images that do not fit into the memory of a single computer. Strong scaling measures how quickly the algorithm can solve a problem of fixed size when it is distributed across an increasing number of computers. We use synthetic images in order to control for variations in the result stemming from image contents. Moreover, synthetic images can easily be scaled to arbitrary size, as required for the weak scaling tests.

**Fig 22:**
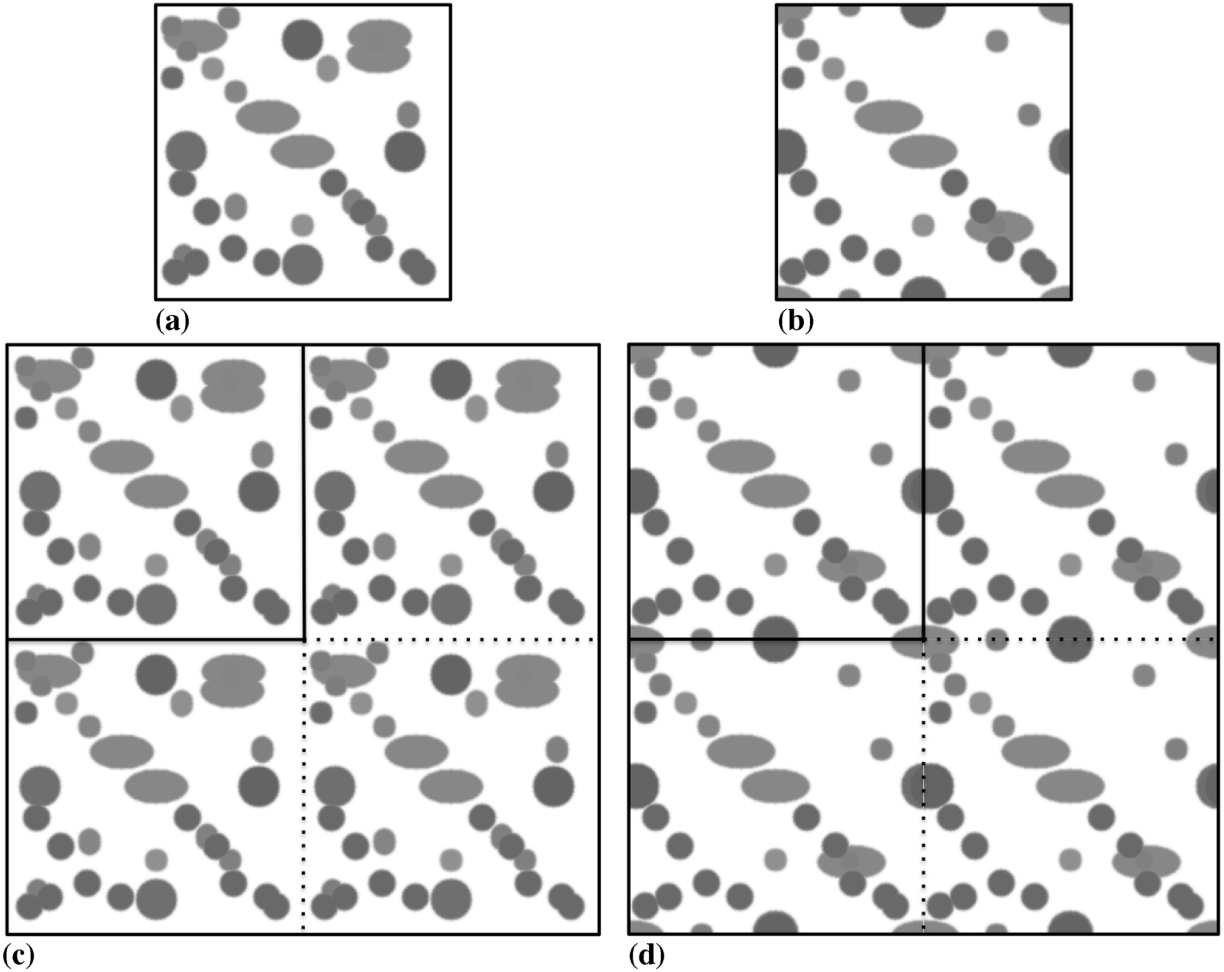
Maximum-intensity projections of the synthetic test images used to assess the parallel performance and scalability of the distributed algorithm. (a) 256 × 256 × 256 pixel unit cell of the first test image where no object touches or crosses the boundary. The image contains 37 ellipsoidal objects of different intensities. All objects are non-overlapping in 3D, even though they may appear overlapping in the maximum projection shown here. (b) 256 × 256 × 256 pixel unit cell of the second test image with objects touching and crossing the boundary. The image contains 48 ellipsoidal objects of different intensities. The object number is higher than in the first image, because some objects are partial, but the fraction of FG pixels vs. BG pixels is the same as in (a) in order to keep the computational cost (i.e., the number of particles) constant. (c) Synthetic workload image of type 1, generated from 4 unit cells by periodically concatenating them. (d) Synthetic workload image of type 2, generated from 4 unit cells by periodically concatenating them.

The two synthetic images used here are shown in Fig 22. These are 3D images, and Fig 22 shows maximum-intensity projections. The top row in Fig 22 shows the “unit cells”, from which the test images are generated by periodic concatenation as shown in the panels below. In the first image (Fig 22a), all objects are local, i.e., there are no objects that cross sub-image boundaries. The second image (Fig 22b) contains objects that cross sub-image boundaries. Comparing the results of the two allows us to estimate the communication overhead from the parallel graph-handling and region labeling algorithms introduced here. In all cases, the algorithm is initialized with circular regions around each local intensity maximum after blurring the image with a Gaussian filter of *σ* = 5 pixel.

In the weak scaling, the workload per processor remains constant, while the overall image size increases proportionally to the number of processors. This way, the workload on 512 processors is an image of 8192 × 4096 × 256 pixels containing 18 944 objects. Periodically repeating the “unit cell” image, rather than scaling it, ensures that the workload on each processor is exactly the same, since every processor locally “sees” the same image.

**Fig 23:**
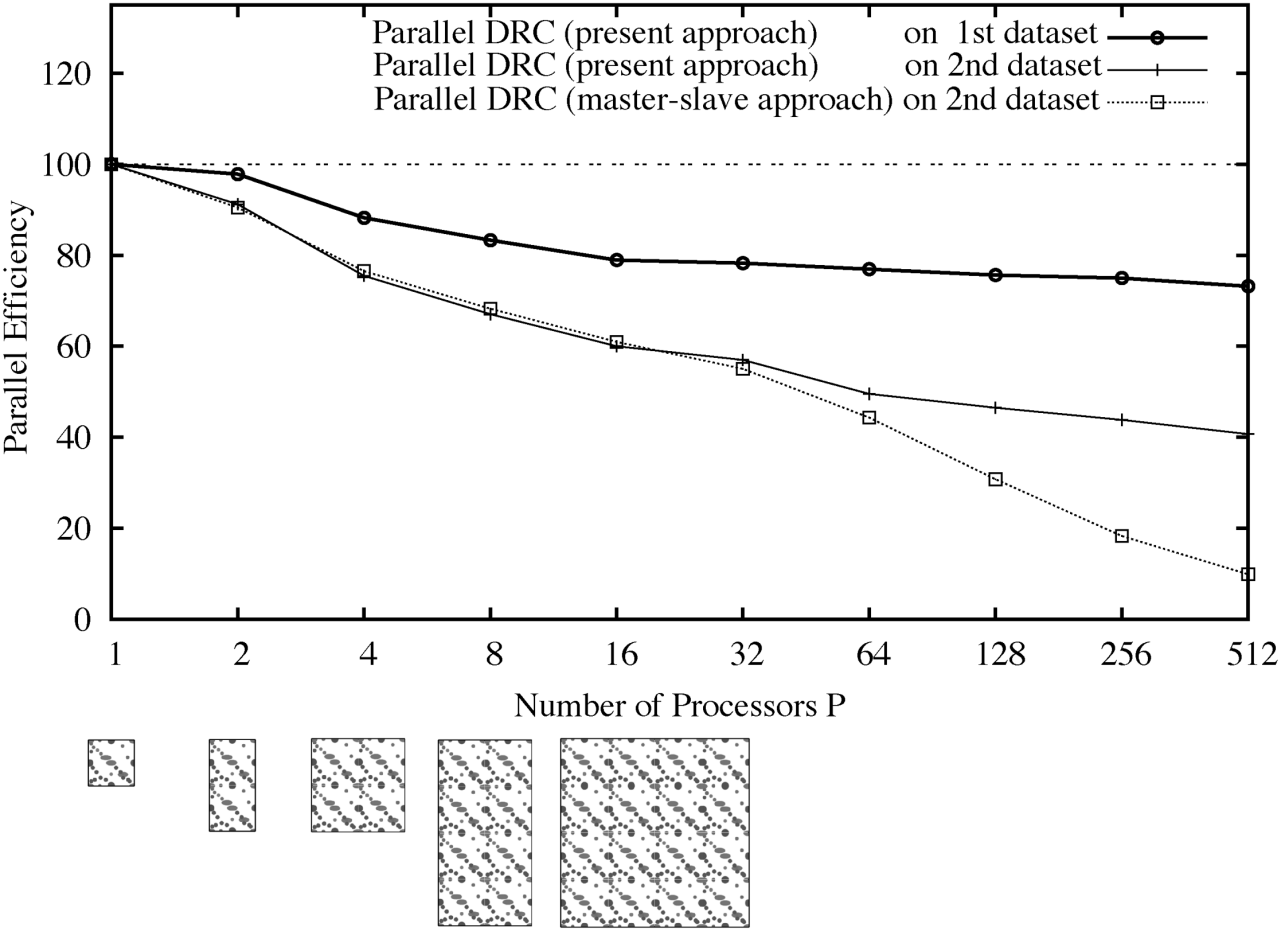
Weak scaling parallel efficiency of the present method in comparison with the classical master/slave approach. Time *t*_1_ is the runtime of the algorithm to process a “unit cell” image on one processor, and *t*_*P*_ is the runtime to segment a *P*-fold larger periodic concatenation image distributed over *P* processors. The images on 1 to 16 processors are shown below the abscissa for illustration.

Fig 23 shows the resulting parallel efficiency (weak scaling) for the two test images. For comparison, it also shows the parallel efficiency when using the classical master-slave approach to graph processing (see Fig 7). This approach does not scale, as the parallel efficiency rapidly drops when using more than 32 processors. This results from the communication overhead due to global communication, and from the additional serialization. The present randomized approach scales for both test images.

Segmentation of the second data set using the present parallel approach on 1, 64, and 512 processors took less than 12, 24, and 29 seconds respectively, corresponding to image sizes of 32 MB, 2 GB, and 16 GB, respectively, in this weak-scaling test. Comparing the results for the first test image, where no objects cross sub-image boundaries, with those for the second test image reveals that about half of the communication overhead is due to boundary particles.

**Fig 24:**
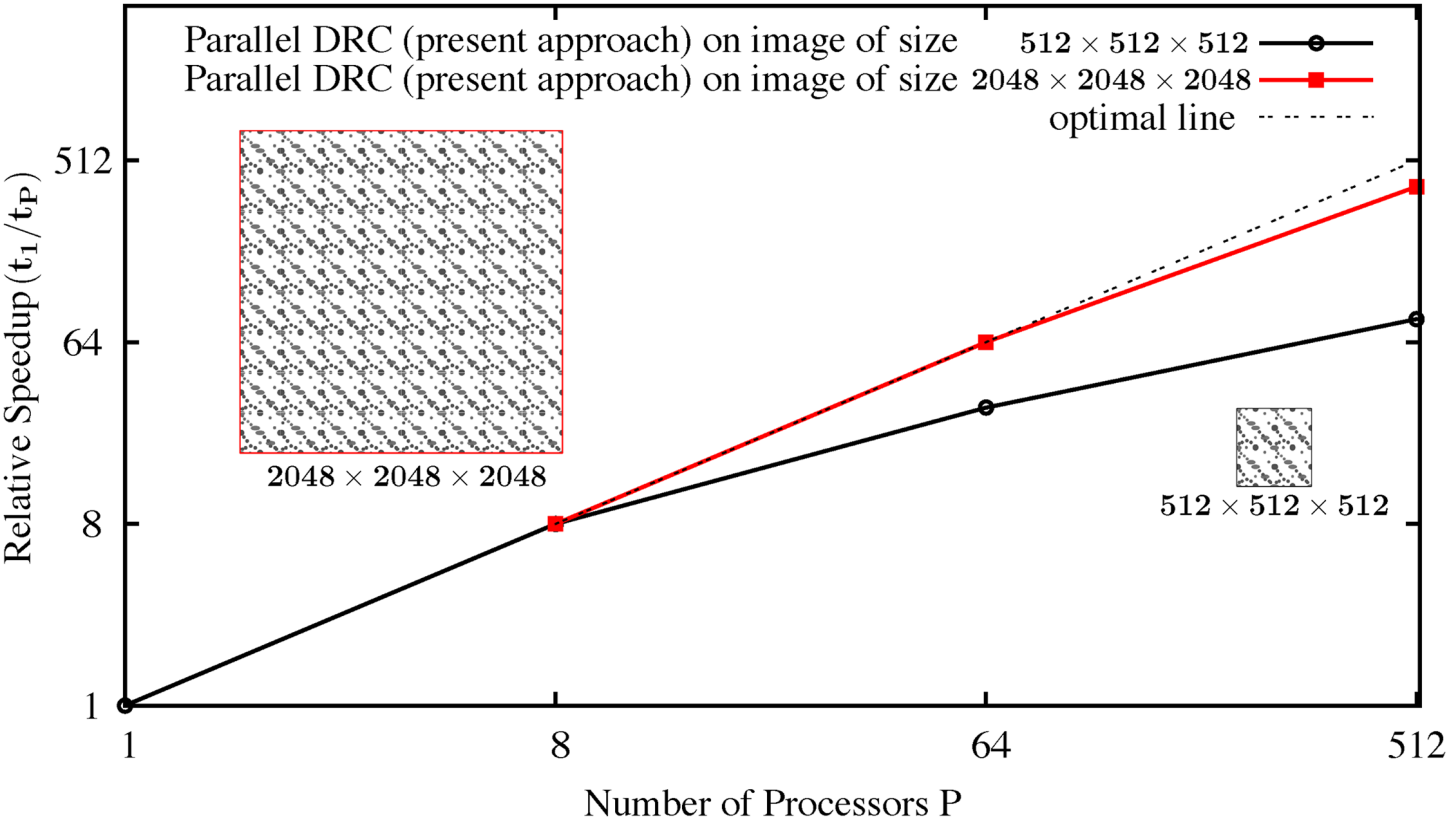
Strong scaling speedup versus number of processors *P* for two different image sizes of 512 × 512 × 512 pixel (black circles) and 2048 × 2048 × 2048 pixel (red squares). The two images are shown in the insets.

Strong scaling measures how efficiently a parallel algorithm reduces the processing time for an image of a given and fixed size by distributing it across an increasing number of processors. Since the workload per processor decreases as the number of processors increases in a strong scaling, the relative communication overhead steadily grows. Strong scalability is hence always limited by problem size with large problems scaling better. We therefore show tests for two different image sizes: a moderate image size of 512 × 512 × 512 pixels (black circles in Fig 24) and a large image of 2048 × 2048 × 2048 pixels (red squares in Fig 24).

For the first image of size 512 × 512 × 512 pixel, the decrease in efficiency beyond 8 processors is due to communication between the processors, which increases relatively to the smaller and smaller computational load per processor. A 30-fold speedup is achieved for this image size on 64 processors. On 512 processors, every processor only has a sub-image of size 64 × 64 × 64 pixel with ghost layers of width 5 pixel all around. Segmentation of this image on 8, 64, and 512 processors took 16, 4.2, and 1.6 seconds, respectively.

For the larger image of size 2048 × 2048 × 2048 pixel, segmentation on one processor is not possible, since it would require 62 GB of main memory. On 8 and more processors, segmentation becomes feasible and takes 6870 seconds on 8 processors. On 64 and 512 processors, the result is computed in 860 and 145 seconds, respectively. A 48-fold speed is achieved on 512 processors relative to 8 processors, which corresponds to a scalability close to the optimal line.

### Application to acquisition-rate segmentation of 3D light-sheet microscopy data

We present case studies applying the present algorithm to segmenting 3D image data from light-sheet microscopy, demonstrating that acquisition-rate segmentation is possible. We use images of stained nuclei and of vasculature in order to demonstrate the flexibility of the method to segment different shapes.

**Fig 25:**
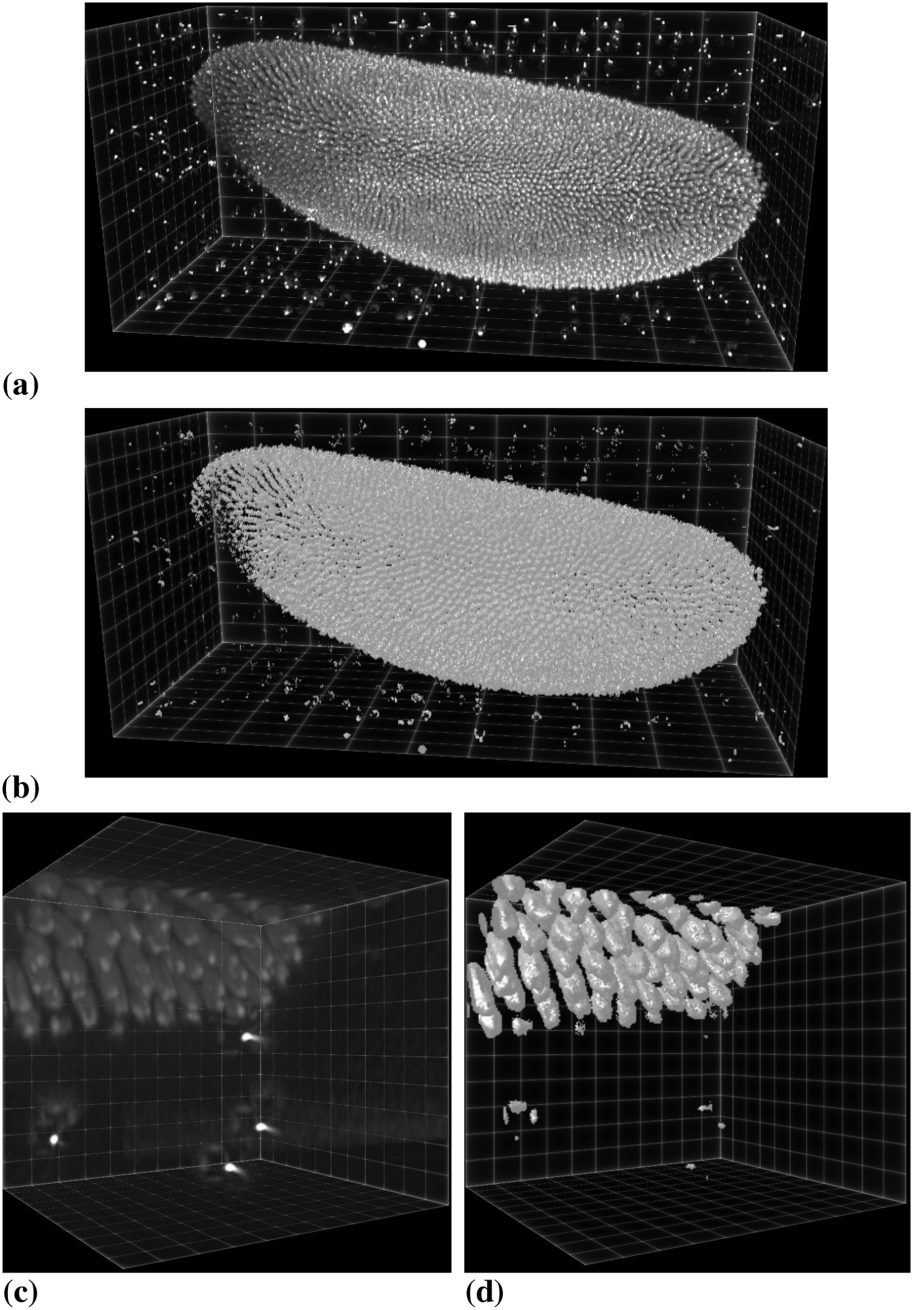
Application of the present implementation to acquisition-rate segmentation of a 3D light-sheet microscopy image using a piecewise constant (PC) image model. All 3D visualizations were done using *ClearVolume* [39]. (a) Raw image showing a *Drosophila melanogaster* embryo at cellular blastoderm stage with fluorescent histone marker (image: Dr. Pavel Tomancak, MPI-CBG). In addition to the nuclei, there are fluorescent beads embedded around the sample as fiducial markers for multi-view fusion and registration [3]. (b) Segmentation result using the present distributed implementation of DRC with the PC image model distributed across 128 processors. The total time to compute the segmentation was 60 seconds, while the microscope acquired a 3D image every 90 seconds. (c) Example of a sub-image from one of the processors. (d) Corresponding part of the segmentation as computed by that processor.

The first image shows a whole live *Drosophila melanogaster* embryo at cellular blastoderm stage with nuclei labeled by a histone marker. This data was acquired on an OpenSPIM microscope [38] in the Tomancak lab at MPI-CBG. The size of the original image file is 4.6 GB at 32 bit depth. The image has 1824 × 834 × 809 pixels. During the segmentation, a total of about 64 GB of main memory is required for DRC. Distributed across 128 processors, this is 500 MB per processor, which fits the memory of the individual cores. The segmentation results using the present distributed algorithm with the PC image model on 128 processors is shown in Fig 25c.

Fig 25e shows the sub-image from one of the processors, and Fig 25f the corresponding part of the segmentation result. Communication across sub-image boundaries ensures that the processors collectively solve the global problem without storing all of it.

Segmenting this image distributed across 128 processors took less than 60 seconds, which is shorter than the time of 90 seconds until the microscope acquires the next time point. We hence achieve acquisition-rate image segmentation in this example, using a state-of-the-art model-based segmentation algorithm that produces high-quality results. If necessary, more processors can be used to further reduce processing time, as we have shown the present implementation to scale well up to 512 processors.

We compare our approach with the TWANG [10] pipeline on 14 cores of one compute node (TWANG does shared-memory multi-threading). TWANG [10] required 24 minutes to compute the segmentation using the 14 cores, which does not allow acquisition-rate processing. The comparison is mainly in terms of computational performance, since TWANG was optimized for segmenting spherical objects, whereas the nuclei in our image are rather elongated. The result from DRC hence shows better segmentation quality.

**Fig 26:**
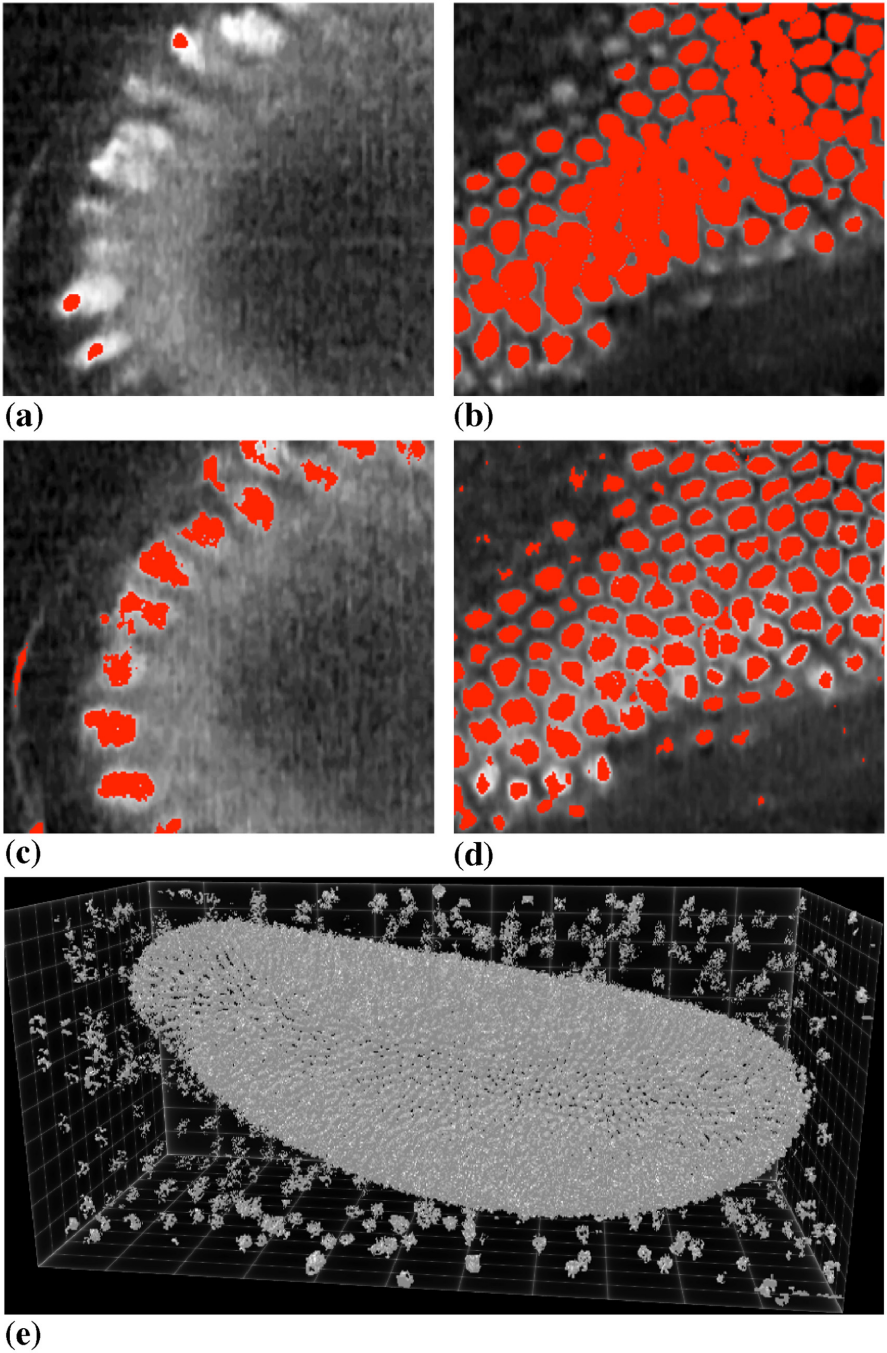
Comparison of the piecewise constant (PC) and piecewise smooth (PS) image models. All visualizations were done using *ClearVolume* [39]. (a) Segmentation (red) overlay in a low-intensity region using the PC model. (b) Segmentation (red) overlay in a high-intensity region using the PC model. (c) Segmentation (red) overlay in the same low-intensity region using the PS model. (d) Segmentation (red) overlay in the same high-intensity region using the PS model. (e) Complete result using the PS model on 128 processors. The total processing time was 250 seconds for this case.

Due to the inhomogeneous fluorescence intensity across the sample, the PC segmentation shown in Fig 25c misses some of the nuclei at the left tip of the embryo. This can be improved using the PS image model instead, which allows for intensity gradients within regions, in particular within the background region. This is shown in Fig 26. Fig 26a shows a low-intensity region where the PC model misses some nuclei. Fig 26b shows a high-intensity regions where the PC model fuses several nuclei together. The corresponding results when using the PS image model are shown in the panels below, in Fig 26c and 26d. The whole-image result when using the PS image model is shown in Fig 26e. The PS model improves the segmentation since it adjusts to local intensity variations in the objects and the background, which is also why it captures more of the fiducial beads around the embryo. This demonstrates the flexibility of DRC to accommodate for different image models, enabling application-specific segmentations that include prior knowledge about the image. The segmentation quality can further be improved by including shape priors [23, 40], as has been demonstrated for DRC [41], or by using Sobolev gradients for which DRC is uniquely suited [42].

Using the PS image model, however, is computationally more involved than using the PC model. The segmentation shown in Fig 26e required 250 seconds to be computed on 128 processors. Acquisition-rate processing using the PS model hence requires about 512 processors.

**Fig 27:**
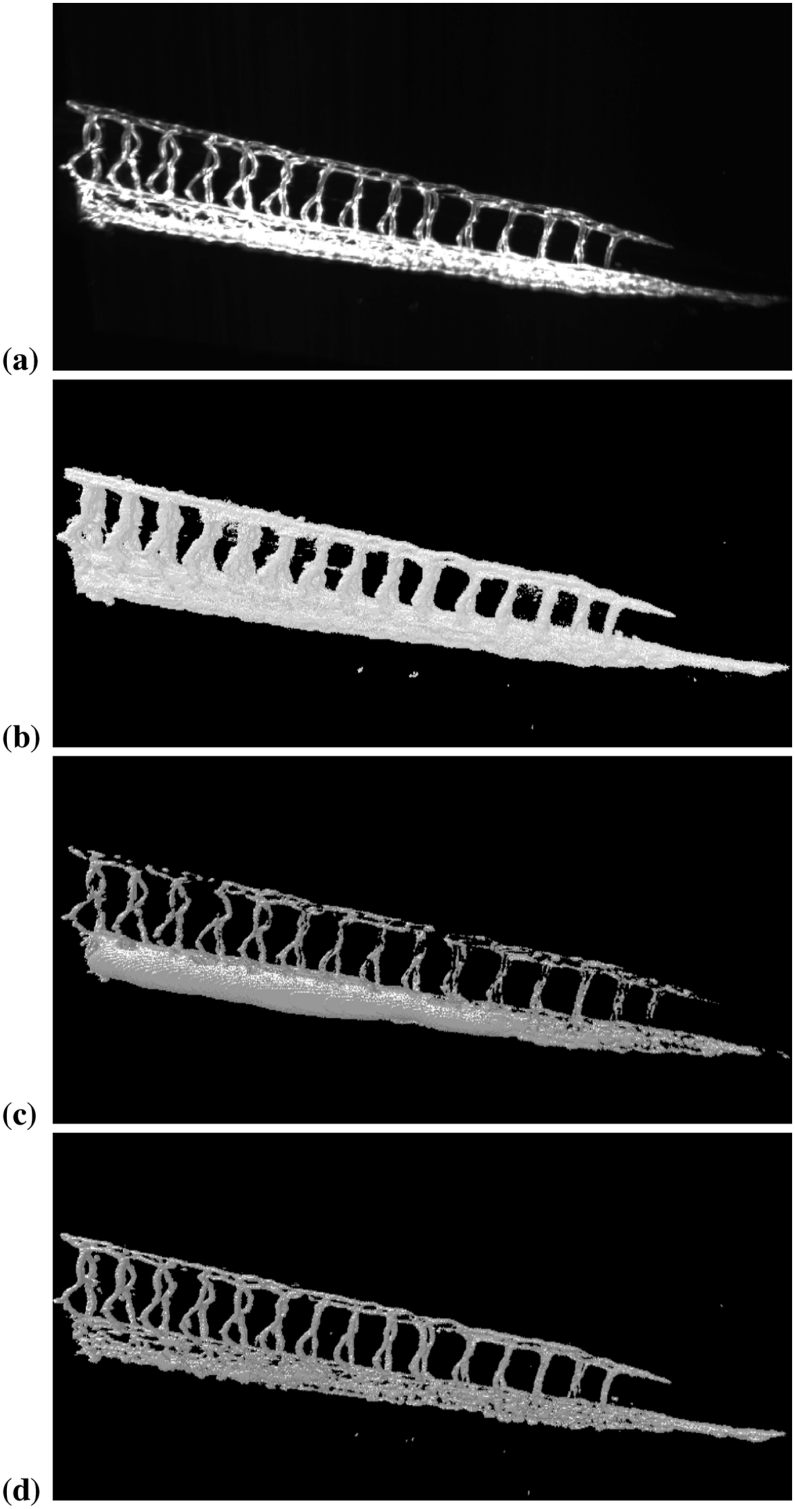
Application of the present method to segmenting zebrafish vasculature in a light-sheet microscopy image. All visualizations were done using *ClearVolume* [39]. (a) Raw image showing the tail part of the vasculature in a developing zebrafish embryo 3.5 days post fertilization (image: Stephan Daetwyler, Huisken lab, MPI-CBG). (b) Initialization using Li thresholding [43]. (c) Segmentation result using the PS image model with Gaussian noise model on 32 processors. The total processing time was 248 seconds. (d) Segmentation result using the PS image model with Poisson noise model [45] on 32 processors. The total processing time was less than 200 seconds.

The second image shows the tail of a live zebrafish embryo 3.5 days post fertilization with the vasculature fluorescently labeled by expressing GFP in endothelial cells (Tg(flk1:EGFP)s843). This image was acquired by the Huisken lab at MPI-CBG using a state-of-the-art light-sheet microscope [1]. The geometric structure of a vascular network is very different from blob-like nuclei, illustrating the flexibility of DRC to segment arbitrary shapes. This image is intractable for specialized blob-segmentation pipelines like TWANG [10]. Fig 27a shows the raw data. The image has 1626 × 988 × 219 pixels. Fig 27b shows the segmentation result using Li’s minimum cross-entropy thresholding [43], as implemented in the software package *Fiji* [44]. We use this thresholding as an initialization for our method. Fig 27c is the segmentation result using the PS image model with a Gaussian noise model. Distributed processing on 32 processors took 248 seconds. In this segmentation, some vessels appear non-contiguous and the caudal vessels (caudal artery and caudal vein) are not properly resolved. This changes when replacing the Gaussian noise model with a Poisson noise model [45], as shown in Fig 27d. Using the correct noise model clearly improves the result, providing further illustration that flexible frameworks like DRC are important. The result in Fig 27d was obtained on 32 processors in less than 200 seconds.

## Discussion

We have presented a distributed-memory parallel implementation of the Discrete Region Competition (DRC) algorithm [15] for image segmentation. Efficient parallelization was made possible by a novel parallel independent sub-graph algorithm, as well as optimizations to the parallel connected-component labeling algorithm. The final algorithm was implemented using the PPM library [17–19] as an efficient middleware for parallel particle-mesh methods. The parallel implementation includes both piecewise constant (PC) and piecewise smooth (PS) image models; it is open-source and freely available from the web page of the MOSAIC Group and from github: https://github.com/yafshar/PPM_RC.

The distributed-memory scalability of the presented approach effectively overcomes the memory and runtime limitations of a single computer. None of the computers or processors over which a task is distributed needs to store the entire image. This allows segmenting very large images. The largest synthetic image considered here had 1.7 ⋅ 10^10^ pixels, corresponding to 32 GB of uncompressed memory. A real-world light-sheet microscopy image of 1824 × 834 × 809 pixels (amounting to 4.6 GB of uncompressed memory) was segmented in under 60 seconds when distributed across 128 processors. This was less than the 90 seconds until the microscope acquired the next time point, hence providing online, acquisition-rate image analysis. This is a prerequisite for smart microscopes [5] and also enables interactive experiments.

We have demonstrated the parallel efficiency and scalability of the present implementation using synthetic images that can be scaled to arbitrary size. We have further reproduced the benchmark cases from the original DRC paper [15] and shown that the parallel implementation produces the same or very close results as the original sequential reference implementation. Small differences may occur, but are limited to isolated oscillatory pixels, which are due to local oscillation detection. This local detection is preferable because it avoids global communication and improves parallel scalability with respect to the traditional master/slave approach.

Although our performance figures are encouraging, there is still room for further improvements. One idea could be to compress the particle and image data before communication. This would effectively reduce the communication overhead and improve scalability. Furthermore, spatially adaptive domain decompositions and dynamic load balancing could be used to reduce load imbalance. Depending on the image contents, not all processors may have an equal amount of particles. This causes asynchronous waiting times that may limit scalability. Due to the checkerboard decomposition used in the graph handling, however, the present implementation is limited to Cartesian domain decompositions, while spatially adaptive trees might be better. Lastly, the local evaluation of energy differences for all possible particle moves can be accelerated by taking advantage of multi-threading and graphics processing units (GPUs). This is possible for DRC, as has already been shown [46], suggesting that processing could be further accelerated by a factor of 10 to 30, depending on the image model.

This leaves ample opportunities for further reducing processing times as required by the microscopy application. Already the present implementation, however, illustrates the algorithmic concept, which is based on randomized graph decomposition and hybrid particle-mesh methods. This enables acquisition-rate segmentation of 3D fluorescence microscopy images using different image models, opening the door for smart microscopes and interactive, feedback-controlled experiments.
